# Association mapping by aerial drone reveals 213 genetic associations for *Sorghum bicolor* biomass traits under drought

**DOI:** 10.1186/s12864-018-5055-5

**Published:** 2018-09-17

**Authors:** Jennifer E. Spindel, Jeffery Dahlberg, Matthew Colgan, Joy Hollingsworth, Julie Sievert, Scott H. Staggenborg, Robert Hutmacher, Christer Jansson, John P. Vogel

**Affiliations:** 1 DOE Joint Genome Institute, 2800 Mitchell Dr, Walnut Creek, CA 94598 USA; 2 UC-ANR Kearney Agricultural Research and Extension Center, 9240 S Riverbend Ave, Parlier, CA 93648 USA; 3 Blue River Technology, 575 N Pastoria Ave, Sunnyvale, CA 94085 USA; 4 Chromatin Inc, Lubbock, TX USA; 5 UC-ANR West Side Research & Extension Center, 17353 W Oakland Ave, Five Points, CA 93624 USA; 6 Pacific Northwest National Laboratory: Environmental Molecular Sciences Laboratory, 902 Battelle Blvd, Richland, WA 99354 USA; 7 Present Address: Monsanto, St. Louis, MO USA

**Keywords:** Sorghum, GWAS, Drought, Drone, Phenomics, Biomass

## Abstract

**Background:**

*Sorghum bicolor* is the fifth most commonly grown cereal worldwide and is remarkable for its drought and abiotic stress tolerance. For these reasons and the large size of biomass varieties, it has been proposed as a bioenergy crop. However, little is known about the genes underlying sorghum’s abiotic stress tolerance and biomass yield.

**Results:**

To uncover the genetic basis of drought tolerance in sorghum at a genome-wide level, we undertook a high-density phenomics genome wide association study (GWAS) in which 648 diverse sorghum lines were phenotyped at two locations in California once per week by drone over the course of a growing season. Biomass, height, and leaf area were measured by drone for individual field plots, subjected to two drought treatments and a well-watered control. The resulting dataset of ~ 171,000 phenotypic data-points was analyzed along with 183,989 genotype by sequence markers to reveal 213 high-quality, replicated, and conserved GWAS associations.

**Conclusions:**

The genomic intervals defined by the associations include many strong candidate genes, including those encoding heat shock proteins, antifreeze proteins, and other domains recognized as important to plant stress responses. The markers identified by our study can be used for marker assisted selection for drought tolerance and biomass. In addition, our results are a significant step toward identifying specific sorghum genes controlling drought tolerance and biomass yield.

**Electronic supplementary material:**

The online version of this article (10.1186/s12864-018-5055-5) contains supplementary material, which is available to authorized users.

## Background

The plant and agricultural research community faces a grave challenge: in a mere three decades, we must reinvent agriculture to feed a growing global population, in an environmentally sustainable manner, while dealing with a projected increase in drought events [1–3]. Sorghum [*Sorghum bicolor* (L.) Moench] could be bred to help address these challenges. Sorghum is the fifth most commonly grown cereal crop worldwide, and over half a billion people rely on it as a daily food staple. It is already essential to food security, as it can grow across a wide range of marginal climates, including regions too hot and dry to grow rice, corn, or wheat. Sorghum has also generated interest in recent years as a bioenergy crop because it can produce exceptionally large biomass yields on marginal lands with limited inputs [4–6].

In order to efficiently develop sorghum biomass varieties, several important research questions must be addressed. First, we must understand the genetic underpinnings of terminal biomass in sorghum and identify specific genes or genetic regions that can be targeted for breeding and engineering efforts. Second, if bioenergy crops are to be compatible with environmental stewardship and increased food production they cannot compete with food crops for productive croplands – they must be grown on underutilized, marginal lands. So, it is also necessary to gain a mechanistic understanding of stress tolerance. Unfortunately, our current understanding of the genetics underlying biomass and/or drought tolerance in sorghum is limited. While there have been many linkage mapping studies for drought tolerance quantitative trait loci (QTLs), there has been almost no validation of causal polymorphisms, which is a crucial step before results are of use to breeders, and the QTL regions identified in these linkage studies have been too large to identify meaningful candidate alleles [7–13]. A genome wide association study (GWAS), by contrast, identifies linkages between SNPs and causal polymorphism based on historical recombination events in a diversity panel, and thus allows for a much finer resolution around the site of a causal polymorphism, while, at the same time, assaying more diversity than a linkage mapping in a bi-parental family. Thus, despite the many QTLs identified for drought tolerance in sorghum over the years, a well-designed GWAS for drought tolerance is needed to identify better candidates for causal polymorphisms underlying the remarkable drought tolerant characteristics of sorghum.

By far, the best studied traits in sorghum are flowering time and plant height, in large part because these traits were the targets of the US Sorghum Conversion Program (SCP). Most sorghum varieties are photoperiod sensitive and do not flower at temperate latitudes, so in 1963, the SCP was begun with the goal of introgressing photoperiod insensitivity and dwarfing alleles into exotic sorghum backgrounds to produce what are now commonly referred to as converted lines or ‘SC’ lines [14–16]. When photoperiod sensitive sorghums are grown in the US, they have greater potential as biomass lines than SC lines because they never transition out of the vegetative stage and so grow all season long. They also do not generally express the genes for short stature, which the converted lines were bred to favor, and which for obvious reasons, also dramatically decrease biomass [5].

In sorghum plant height is thought to be primarily determined by four dwarfing loci, *Dw1*-*Dw4,* all of which have been cloned with the exception of *Dw4* [17]. Flowering time is likewise thought to be controlled by six major loci (*Ma1*-*Ma6*), of which *Ma1* and *Ma3* have been cloned [14, 16, 18]. Total/terminal plant biomass, however, is a complex trait that is affected by many genetic factors beyond plant height and flowering time. While a few recent GWAS have attempted to map single nucleotide polymorphisms (SNPs) linked to causal polymorphisms of sorghum biomass traits, very few candidates have been identified [19–22]. One of the main reasons for this is the difficulty of measuring biomass in the field. The most common method of measuring plant biomass is destructive, which means that only a single measurement can be taken per plot for an entire growing season, an under-powered approach for a GWAS. Attempting to collect precise non-destructive plant growth measurements (e.g. height, leaf area index) by hand over the course of the growing season, however, is highly impractical. Many others have pointed out that the bottleneck for the modern GWAS is no longer the genotyping, which is now relatively inexpensive and almost entirely automated, but the phenotyping. So, we must solve this new problem the same way we solved the first – with the development and application of new technology, generally referred to as ‘phenomics’ [23–25].

Approaches to field-based plant phenomics include gantry systems, ground-based vehicles, and aerial drones. With all phenomic strategies, however, the goals are high-throughput, non-invasiveness, accuracy, and automation. Gantry systems are able to lift large, imaging sensor suites, making them ideal for engineering research and development, but are fixed installations and cover a small area compared to the needs of commercial plant breeders. Ground-based vehicles, including high-clearance tractors and slim-profile autonomous robots which maneuver between crop rows, can measure traits that are challenging to observe from above, but can be physically impeded by downed plants, tall plants, and tillering, particularly in sorghum. Aerial drones, by contrast, can cover large areas quickly, allowing all genotypes in a study to be measured simultaneously, and are not impeded by plant height, which allows them to capture data throughout the entire growth season [26]. Regardless of platform, however, the most challenging aspect of any phenomic approach is the data analysis and conversion of imagery and sensor data into trait measurements that are relevant to plant breeders.

We present here the results of a GWAS in sorghum in which all of the phenotype data, including total fresh biomass (BWET), biomass at 65% moisture content (B65), leaf area index (LAI), and plant height (PH), were collected by aerial drone and ground-truthed, once per week, over the course of the growing season. The panel included 620 diverse public sorghum conversion (SC) lines and 26 proprietary sorghum hybrids provided by Chromatin Inc. To investigate the interaction between biomass and drought, as well as to better understand the genetic architecture of drought tolerance in sorghum, the panel was planted in three treatment plots including a control, pre-flowering drought stress, and post-flowering drought stress plots, at two locations in central California. We thus refer to the resulting phenotype dataset as ‘high density’, and the GWAS we performed as a ‘high-density phenotype’ GWAS, or an ‘HDP-GWAS’. Using a custom HDP-GWAS data analysis pipeline and novel data visualization tools, we identified 213 highly reliable GWAS peaks, a number of significant and reliable associations far larger than in any previously published GWAS performed in sorghum.

## Results and discussion

### Germplasm selection, genotyping and population structure

Six hundred fourty-eight lines including 620 SC lines, two inbred lines with known drought tolerance and 26 hybrids were selected to make up the diversity panel to assay the natural diversity of *Sorghum bicolor* (Additional file 1). The SC lines were selected to represent the broad genetic and environmental base from which sorghum was domesticated and include representatives of all major sorghum morphology types (a.k.a. races -- bicolor, caudatum, guinea, kafir, and durra) and a range of breeder ‘working groups’ currently recognized in traditional sorghum breeding classification schemes. Converted sorghum lines were selected to form the bulk of the panel based on the hypothesis that transition to flowering and the shift to reproductive growth stages have profound effects on genetic regulation of most, if not all traits, and that performing a GWAS in lines that did not flower (i.e., a panel of non-converted lines) would identify fewer polymorphisms linked to biomass and/or drought tolerance.

The panel was genotyped using genotyping-by-sequencing (GBS). Imputation accuracy (FILLIN, methods) was calculated as 99.8% for major allele homozygotes, 92.9% for minor allele homozygotes, and 55.6% for heterozygotes. After filtering for bi-allelic SNPs with call rates ≥75%, 183,989 SNPs remained with an overall heterozygosity rate of 1.34%. When the hybrid lines were removed, only ~ 1% of SNP calls were heterozygous. A final set of 131,544 SNPs with minor allele frequency (MAF) ≥ 0.01 were used for all GWAS analyses. Two individuals with genotyping rates < 60% were dropped from all subsequent analyses, after which the mean call rate per genotype was 91.5%.

**Fig. 1:**
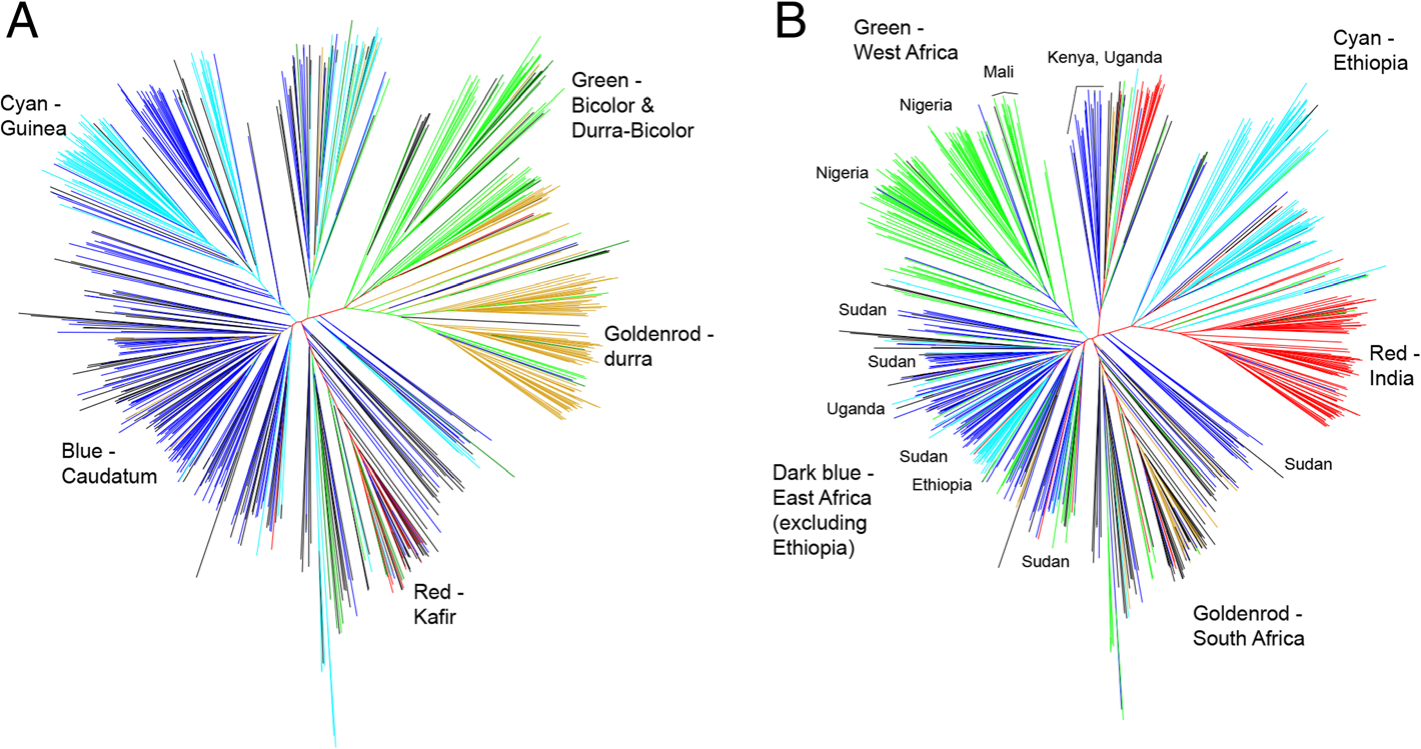
Genetic relationship between lines. Neighbor joining trees for 648 diverse *Sorghum bicolor* lines including 622 inbred sorghum lines and 26 proprietary Chromatin Inc. hybrids colored by morphology type (**a**) and country of origin of exotic parents (**b**). Together, morphology type and country of origin explain most of the genetic structure of this sorghum panel

Neighbor-joining (NJ) tree analysis, principle components analysis (PCA) analysis, and partitioning around k-medoids analysis (PAMK) each identified five subpopulation groups within the diversity panel (Fig. 1, Additional file 2: Figure S1). The NJ tree analysis revealed that the genetic structure could be explained by a combination of morphology type and country of origin of the exotic parents used in the SCP, specifically, West Africa (Nigeria, Mali), East Africa excluding Ethiopia (Sudan, Kenya, and Uganda), Ethiopia, India, and South Africa, in agreement with previous studies of sorghum natural diversity (Fig. 1) [16, 22, 27, 28]. Due to the high degree of confounding between morphology type and country of origin, it was not possible to determine which of these two factors was the primary driver of subpopulation structure in this dataset, however it is clear that the two factors together explain almost all clusters and sub-clusters on the NJ tree well (Fig. 1). The comparison of our tree and PCA results to those published on larger and more extensive analyses of sorghum natural diversity suggest that our panel is representative of sorghum natural diversity [22, 27, 28] (Additional file 1). PAMK also found the number of subpopulations to be 5 by maximum average silhouette width (aws) = 0.082.

**Fig. 2:**
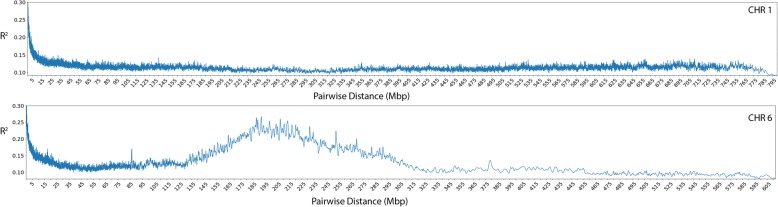
Linkage disequilibrium. Gaussian kernel smoothed pairwise linkage disequilibrium (LD), r^2^, by SNP pair distance (bp) for chromosomes 1 and 6. Pairwise LD was calculated for all pairs of SNPs on each chromosome using Plinkv1.9, and a Gaussian kernel smoother (σ = 500) fit to model the relationship between SNP distance and pairwise LD on each chromosome. Most chromosomes resembled chromosome 1 (top) in that LD quickly decayed to a baseline of ~ 0.1 r^2^. Several chromosomes such as chromosome 6 (bottom), however, were subject to large linkage blocks as a result of linkage drag around conversion loci on these chromosomes. Similar plots for the other chromosomes can be found in Additional file 2: Figure S2

Pairwise linkage disequilibrium (LD, r^2^) was calculated for all SNPs on each chromosome. For each pair of SNPs, the relationship between pairwise distance and r^2^ was then calculated using a Gaussian kernel smoother (σ = 500). Across all chromosomes, this yielded an average baseline LD (r^2^) of 0.12 (Fig. 2, Additional file 2: Figure S2, Additional file 3). LD decayed to less than 0.3 r^2^ by an average of 39.7 Kb, however it is important to note that regions of substantially higher LD were identified across the genome, most notably, on chromosomes with known conversion loci, the most extreme of which were found on chromosomes 6 and 9, in the regions of *Ma1*/*Dw2* and *Dw1* respectively (Fig. 2, Additional file 2: Figure S2, Additional file 3) [16]. These areas of high local LD were taken into account when interpreting the results of the GWAS experiments.

### Phenotyping

Plant phenotypic traits were measured by drone in Parlier, CA (KARE) and Five Points, CA (WREC) once per week over 12 weeks during the 2016 growing season. At each location, each genotype was grown under three drought conditions: pre-flowering drought stress (PRE), post-flowering drought stress (POST) and control. The drone measured four plant traits: 1., vegetative biomass adjusted to 65% moisture content (tons/acre, B65), a sorghum industry standard for measuring biomass yield and assessing the amount of bioenergy feedstock a sorghum line can provide, 2., fresh total plant biomass (tons/acre, BWET), 3., plant height (m, PH) and 4., leaf area index (LAI).

For each phenotype and drought stress treatment, drought tolerance was calculated as an additional phenotype by taking the deviation between PH, LAI, B65, and BWET for PRE and POST treatments and the control treatment, respectively, for an additional two derived phenotypes per individual, time-point, and location. Thus, the total number of drone phenotype data-points was 171,072 for a high-density phenomics (HDP) dataset. In addition to the phenotype data collected by drone, field-based phenotypes were collected for flowering time at KARE, and drone-collected photos were used to determine flowering time for WREC.

### Phenotype data filtering

Prior to running GWAS, a phenotype data filtering method was developed and applied to the drone-collected phenotype data to reduce noise and remove outliers. Briefly, for each individual, trait, treatment, and location combination, the drone measurements were plotted versus time. A kernel smoothing line was then fit for each plot, and outliers were identified based on the size of the residual of a given data point from the fitted spline (Additional file 2: Figure S3). Using this method, 2.2% of the PH data points, 5.3% of the LAI data points, 5.9% of the B65 data points, and 7.7% of the BWET data points were removed from the dataset, and treated as missing data for all subsequent GWAS analyses. Our filtering method was found to be highly robust for removal of outliers based on the revised set of time-plots produced after outlier filtering, as well as the high quality of the GWAS results (Additional file 2: Figure S4). The resulting phenotype dataset was also used for calculating the derived drought tolerance phenotypes.

Post-phenotype data filtering, all four phenotypes and treatments were well correlated within a given site and time-point, as was expected based on the general proportionality of different measures of plant biomass, although LAI was slightly less well correlated than the other three traits with the group as a whole (Additional file 2: Figure S5). All filtered phenotypes followed approximate normal distributions (Additional file 2: Figure S6).

### Drone-based genome-wide association studies

Single-variate GWAS were run using GEMMA for every combination of trait, treatment, time-point, and location for a total of 460 GWAS. For each GWAS, the following covariates were tested for inclusion in the GEMMA model: no covariates, 1 principle component (PC), 2 PCs, 3 PCs, 4 PCs, 1PC + flowering time (FT), 2 PCs + FT, 3 PCs + FT, and 4 PCs + FT. Of these different models, the simplest model resulting in the best QQ-plot was selected for each GWAS. QQ-plots varied widely in terms of adherence to the null hypothesis, suggesting that for some trait by time by treatment by location combinations (TTLs), population structure, some of which was likely caused by linkage with SCP selection loci, was a confounding factor, however this was not the case for all TTLs, suggesting that false positives due to structure may be present in some GWAS, but not in others – the subsequent analysis methods take this into consideration (Additional file 2: Figure S7). Multiple test correction to a false discovery rate of 0.1 was performed for all *p*-values (wald test, or p-wald) for each GWAS.

### Consolidating results of 460 GWAS into conserved, reliable peaks

**Fig. 3:**
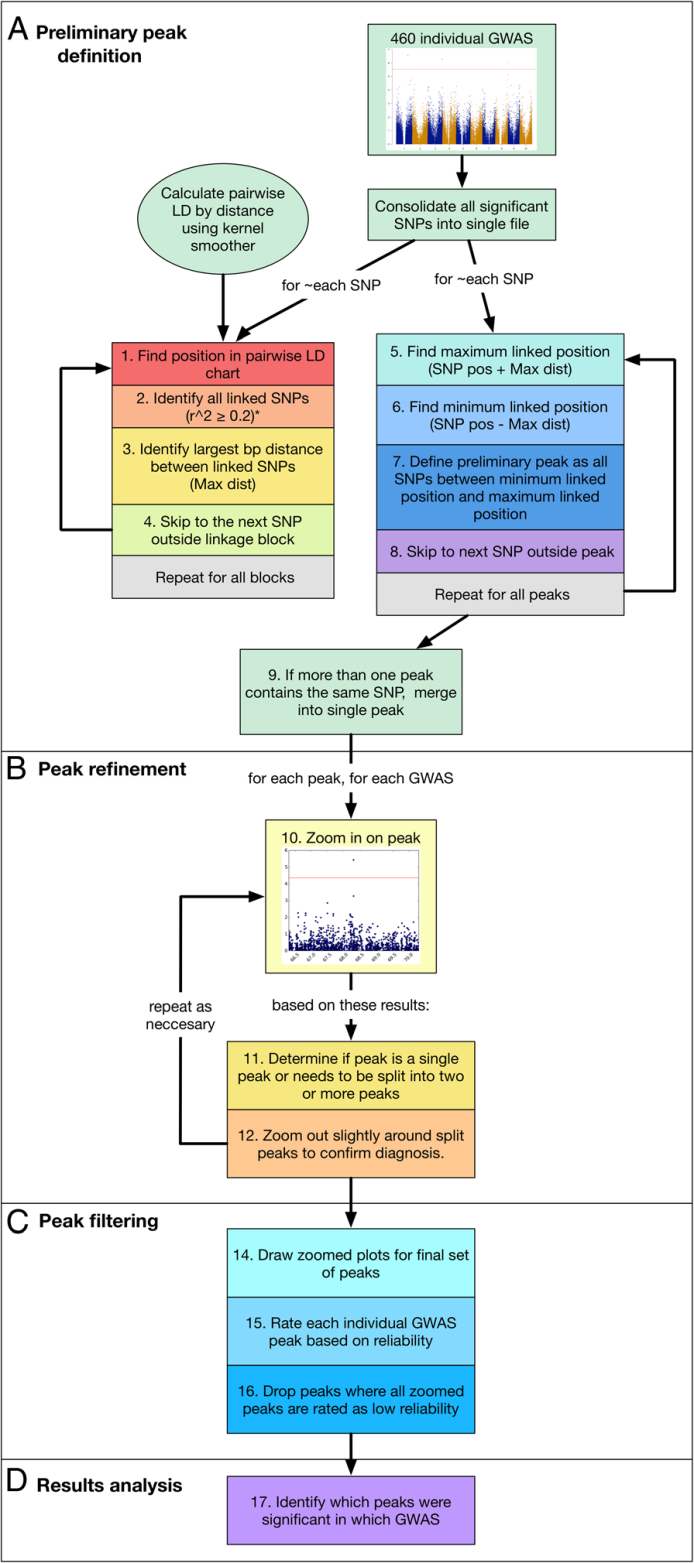
Overview of HDP-GWAS peak definition pipeline. To define preliminary GWAS peaks, all SNPs identified as significant in at least one out of 460 individual GWAS were consolidated into a single file. Local pairwise LD (r^2^) was calculated by first calculating the relationship between pairwise LD and SNP pair distance using a Gaussian kernel smoother (σ = 500), after which, for every SNP in a particular linkage block, the SNP position was found in the pairwise LD table and all linked SNPs identified (**a**, 1–2). A SNP was considered linked if r^2^ ≥ 0.2 for all chromosomes except chromosomes 6 and 9, for which a SNP was considered linked if r^2^ ≥ 0.3. (**a**, 2). Max distance (Max dist) was then defined as the largest bp distance between linked SNPs (**a**, 4). This process was repeated for all linkage blocks. Once max dist was defined for each significant SNP, the upper boundary of each preliminary GWAS peak could be defined as SNP position + max dist, and the lower boundary of each GWAS peak could be defined as SNP position – max dist (**a** 5–7). All SNPs falling in between the boundaries were then considered to be within the same GWAS peak (**a**, 8). This process was repeated for all peaks. In the event that more than one peak contained the same SNPs, they were merged into a single peak (**a**, 9). After defining preliminary peaks in this manner, peaks were refined by drawing ‘zoomed’ Manhattan plots around peaks, i.e., SNPs +/− 50 Kb from the preliminary peak boundaries (**b**, 10). Each zoomed Manhattan plot was then assessed visually to determine if the peak was, in fact, a single peak, or if the pattern of linkage indicated that the peak should be split into two or more peaks (**b**, 11). If it was determined that a preliminary peak should be split into two or more peaks, the diagnosis was confirmed by drawing second zoomed Manhattan plot including SNPs +/− 2 Mb around the peak boundaries (**b**, 12). After peaks were refined in this way, each individual zoomed Manhattan plot was rated either 1, 2, or 3 based on the evidence suggesting the peak was not an artifact, using visual assessment, where a rating on 1 indicated a peak with no evidence to suggest it was not an artifact, and a rating of 3 indicated a peak with very strong evidence it was not an artifact. All other peaks were rated as 2 (**c**, 14–15). Any GWAS peaks with only ‘1’ ratings were removed from the final set of significant GWAS peaks (**c**, 16). The final step of the pipeline is results analysis, i.e., identifying the combinations of trait, treatment, time point, and location that resulted in each significant GWAS peak (**d**, 17)

The 460 GWAS performed identified a total of 12,014 significant associations after multiple test correction, which corresponded to 3907 unique SNPs. To identify the number of genetic loci we defined conserved and reliable peaks across all GWAS. A conserved peak is defined as a peak that was identified in more than one GWAS. A reliable peak is defined as one that is not likely to be the result of a data artifact such as population structure. The high density GWAS pipeline we developed to define our GWAS peaks is illustrated in Fig. 3 and described in detail in the materials and methods.

Using this pipeline, a total of 213 distinct GWAS peaks were identified. The mean of the highest significant –log (*p*-value) identified per SNP, after FDR correction, was 2.74, ranging from a maximum of 12.86 to the defined minimum of 1. The average distance spanned by the significant SNPs across all GWAS runs was 149.84 Kb, ranging from a minimum of 0 (only one significant SNP) to a maximum of 1.6 Mb (Table 1, Additional files 4 and 5).

**Table 1:** Summary of final GWAS peaks identified using peak definition pipeline

	max p-val	narrow peak span (Kb)	wide peak span (Kb)
mean	2.74	32.8	149.84
std	1.41	61.67	236.13
min	1.01	0	0
25%	1.85	0.22	9.09
50%	2.36	6.31	60.56
75%	3.08	39.9	179.82
max	12.68	437.36	1621.43
count (N peaks)	213		

‘max p-val’ = highest corrected *p*-value for any SNP in a given peak over the 460 individual GWAS runs, narrow peak span (Kb) = smallest distance spanned in Kb by significant SNPs for a given peak over the 460 GWAS runs, where a value of 0 indicates that a single SNP passed the significance threshold, wide peak span (Kb) = Kb range of the union of *all* SNPs that surpassed the significance threshold in *any* GWAS that registered a given peak (i.e., the largest, significant bp position out of all GWAS runs - smallest significant bp position out of all GWAS runs, for a given peak). The mean, standard deviation (std), minimum, maximum, and 25%, 50% and 75% quantiles were calculated across these values for the final set of 213 GWAS peaks to produce the above summary

### Introducing the Manhattan blot: Visualization of many single variate GWAS results

**Fig. 4:**
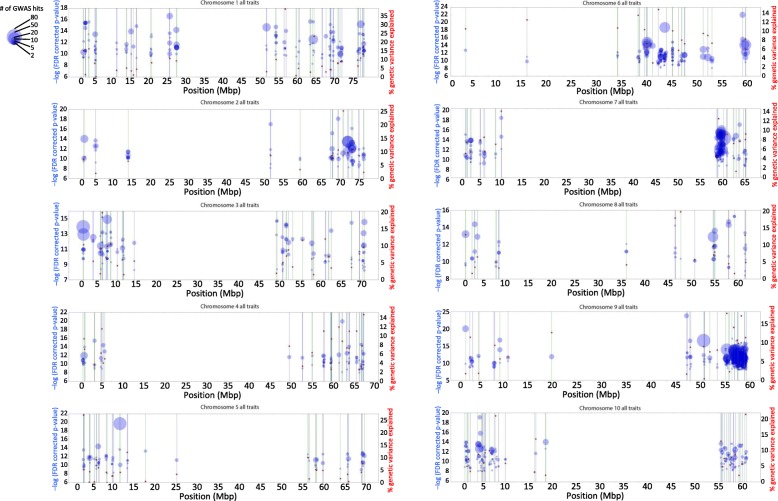
Manhattan blots (M-blots) for chromosomes 1–10. We designed the Manhattan blot as a new method for viewing the results of a large number of single-variate GWAS, as might be performed to utilize HDP-GWAS data -- the results of 460 single-variate GWAS results in the case of the current study. Each point on an M-blot represents a SNP that was significant in at least one of the 460 GWAS where the x-axis is the physical position of the SNP in Mb (by chromosome), the left y-axis gives median of the –log (FDR corrected *p*-value) across all GWAS where the SNP was significant, and where the size of the point is proportional to the number of independent GWAS in which the SNP registered as significant (*N*). These M-blots show the combination of the results for all trait by treatment by time-point by location combinations. The alternating blue and green vertical lines delineate the physical positions of distinct peaks defined using our peak definition pipeline. The right y-axis gives the value of the red star – the highest median percent variance explained (PVE) calculated for the SNPs within the interval of each peak

To jointly visualize the results of 460 GWAS with the goal of identifying the strongest candidates, we designed a variation on the Manhattan plot -- the “Manhattan blot” (M-blot). Like a Manhattan plot, the M-blot x-axis plots the SNP physical position and the y-axis plots the SNP –log (*p*-value), however on an M-blot, only the SNPs that were significant in at least one GWAS are plotted, and *the size of the point is proportional to the number of times the SNP was significant*. The SNPs are plotted on a per chromosome basis, and the exact y-axis position of each point is the median of the –log (FDR corrected *p*-value) across all GWAS where the SNP was significant. The physical boundaries of the GWAS peaks defined using our peak definition pipeline are delineated by alternating blue and green vertical lines (Fig. 4; Additional files 4 and 5).

The assumption at the heart of the M-blot is that the more GWASs that identify a particular SNP as significant, the more likely that SNP is to be linked to a causal polymorphism. This assumption is borrowed from the meta-GWAS approach, which relies on replication between separate experiments to lend reliability to the finding that any given SNP, region, or peak is truly linked to a causal polymorphism. In our experiment, we utilized the related idea that replication across time, environments, and/or treatments can lend similar reliability to our statistical inference.

Since all of our GWAS were performed in the same population (unlike in a traditional meta-GWAS), it was important we minimize the possibility that SNPs identified many times were the result of linkage to a subpopulation structure locus. We did so by removing peaks where all individual GWAS appeared to be the result of population structure artifact (Fig. 3c). The M-blot, itself, also makes it easy to visually assess if this type of subpopulation artifact is present by examining how many circles fall in the physical space of the peak, i.e., in a vertical line. Since each point/circle is a different significant SNP, the more circles that fall in a vertical line, the less likely the peak is an artifact of population structure. The M-blot can thus be interpreted as follows -- the more large circles that fall in a vertical line, the stronger the GWAS peak candidate. The second (right) y-axis on our M-blots plots the highest median percent variance explained (PVE) for a given peak (red stars; Fig. 4; Additional files 4 and 5).

A variation on the M-blot, the Manhattan blot B (M-blot B) was created to further simplify the data presentation (Additional file 2: Figure S8). The M-blot B differs from the M-blot only in that only a single SNP is plotted for each defined GWAS peak – it still shown as a series of blue and green vertical lines. This single SNP is the SNP with the highest median *p*-value of all the SNPs in a given peak. The count of GWASs in which the SNP was identified as significant was then adjusted to equal the sum of the peak SNP counts as well as the sum of counts of all the other SNPs in the peak interval. The point size was then plotted as proportional to this grand sum for each peak. The advantage of the M-blot B is that by glancing only at the single circle, it is easier to gauge the total number of SNPs significant in a given interval across all 460 GWAS (Additional file 2: Figure S8, Additional files 4 and 5).

### Percent phenotypic variance explained and allele effects

Percent phenotypic variance explained (PVE) and phenotypic allele effect were calculated for each individual GWAS, for each SNP that was significant. Given that 460 individual GWAS were performed, these values are more interesting when summarized. PVE was plotted on Manhattan blots as described above, and the mean, median, standard deviation, minimum, and maximum PVE values for each SNP are given in Additional file 6. PVEs ranged from a maximum of 39.3% (S01_56985753) to almost zero. The mean PVE was ~ 5.27%.

**Fig. 5:**
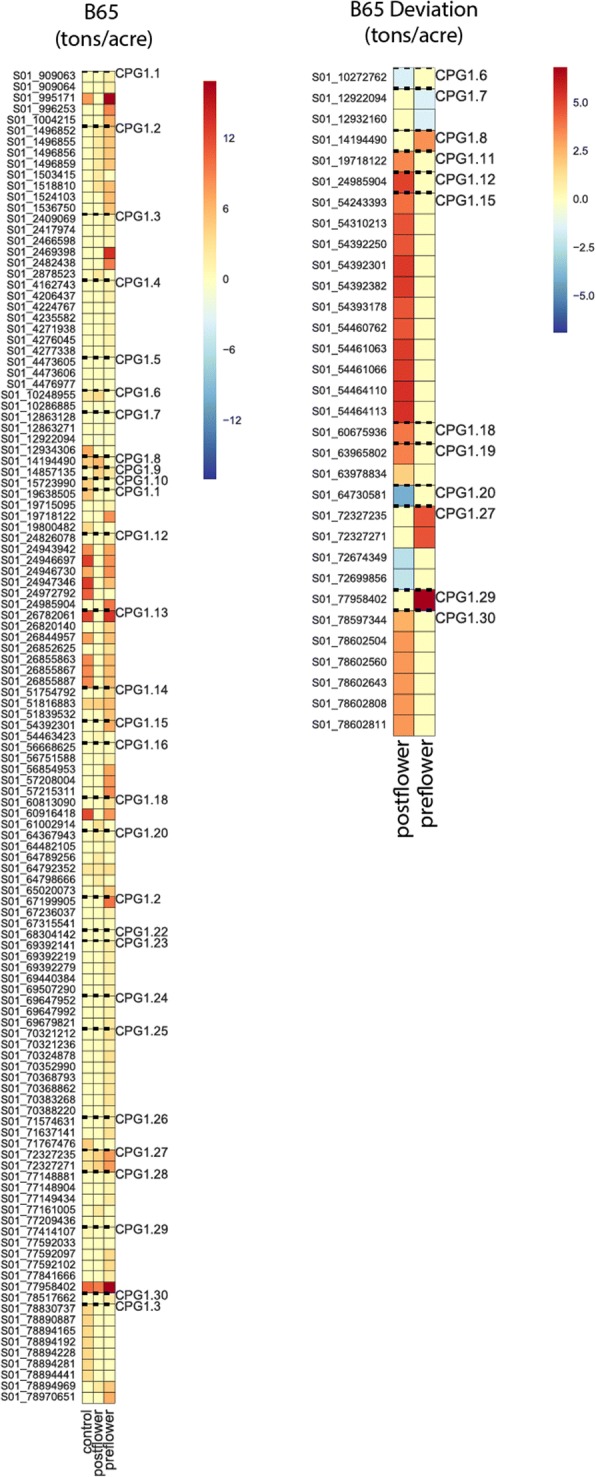
Average allele effects. Heat maps showing the average allele effects across locations and time-points of significant SNPs on chromosome 1 for B65 (left) and B65 deviation (right, where post-flowering = control – post-flowering data and pre-flowering = control – pre-flowering data), for each treatment. Note that each heat map has its own scale, but in all cases, darker red indicates that the minor allele confers an increase in the trait measurement (i.e., increased drought tolerance), darker blue indicates that the minor allele confers a decrease in the trait measure (i.e., decreased drought tolerance), and yellow indicates an effect close to or at 0. SNPs between dashed lines are in the same GWAS peak

For all traits, allele effects of all sizes were identified. Allele effects for B65 ranged from − 1.87 to + 14.81 tons/acre, B65 deviation from − 4.2 to + 11.18 tons/acre, for BWET from − 2.35 to + 22.67 tons/acre, for BWET deviation from − 5.6 to + 16.04 tons/acre, for PH from − 0.08 to + 1.38 m, for PH deviation from − 0.45 to– + 0.61 m, for LAI from − 0.60 to + 1.73 and for LAI deviation, from − 0.72 to + 1.11 (Additional file 7). Some of the very large effect alleles are undoubtedly conversion loci, responsible for flowering time insensitivity and/or dwarfism, however, not all large effect loci overlap with conversion QTL, which suggests promising avenues for future research (Fig. 5, Additional file 2: Figure S9). In general, SNPs where the minor allele *decreased* drought tolerance were only observed when deviation data were used. Far more minor allele effects were observed that increased drought tolerance, suggesting potential for large gains from selection for drought tolerance breeding programs.

### SNP effects by subpopulation

**Fig. 6:**
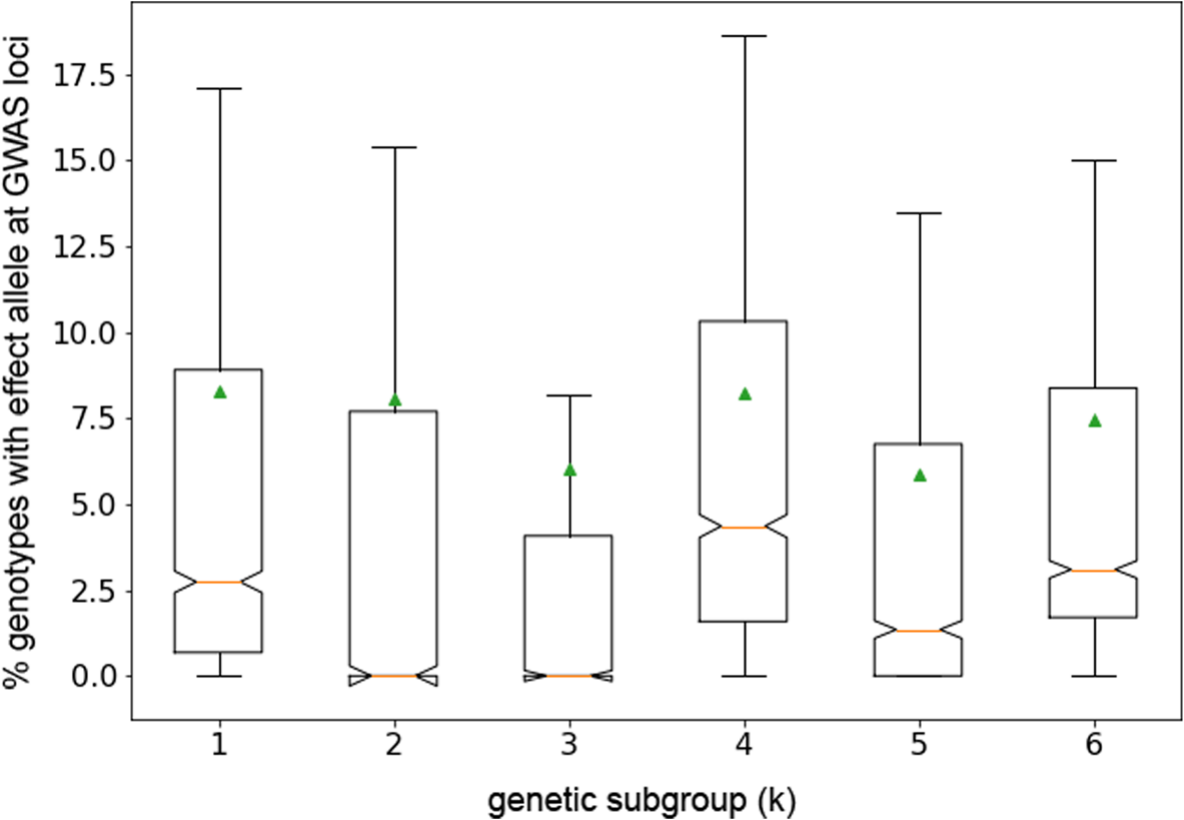
Distribution of effect alleles among genetic subgroups. Average percent of genotypes with effect alleles at GWAS loci by genetic subgroup across 1673 SNPs within 213 GWAS peaks. For each genetic subgroup (K = 1–5), for each SNP, the number and percent genotypes homozygous for the effect (minor) allele was tabulated. The mean, median, range, and interquartile range (IQR) were then calculated for each subgroup. Box whiskers show range, green triangles show mean, lines show median, and box outlines the IQR. The difference between the medians by one-sample ANOVA was highly significant (f = 16.1, *p* = 3.84E-13). Box for group K = 6 shows statistics across all individuals

When one or more subpopulations have been historically untapped by modern breeders, these subpopulations can serve as reservoirs of new, agronomically useful alleles. In order to determine if any such reservoirs exist among the five sorghum subpopulation groups in our panel, we looked at the number of individuals from each subgroup that carried the effect allele for any given GWAS SNP. Fig. 6 shows the relative differences in the percentage of individuals in each subgroup carrying effect alleles over the 213 peaks. The individual percentages are graphed for each peak in Additional file 2: Figure S10. The results of these analyses suggest that, as expected, for most of the peaks, the number of individuals with effect alleles was unevenly distributed across the five genetic subgroups. The peaks that were exceptions to this rule are most likely associated with conversion loci, i.e., the SNPs are probably derived from the recurrent parent in the SCP rather than the exotic parent.

In the dataset as a whole, a median of ~ 3.1% of the genotypes carried minor alleles at loci associated with the 213 GWAS peaks. When this percentage is broken down by subgroup, group K4 is enriched relative to this median, and greatly enriched relative to the other subgroups: ~ 4.4% of K4 genotypes carried minor alleles at effect loci compared to ~ 2.7% of K1 genotypes, ~ 1.4% of K5 genotypes, and ~ 0% of K2 and K3 genotypes, by median (Fig. 6). These differences were highly significant by one-sample ANOVA (F = 16.1, *p* = 3.84E-13).

Group K4 was also found to have the highest number and percentage of private SNPs, meaning that a higher proportion of SNPs with minor allele effects occurring *only* in a single subgroup were found for K4 than any other subgroup. One hundred twenty-two of the 1673 final unique significant SNPs linked to the end set of 213 GWAS peaks were private to K4, ~ 7.3% of the GWAS associated SNPs and ~ 0.5% of all SNP genotypes in K4. K1 had the second most private SNPs, 57, ~ 0.2% of K1 genotype calls. For the other three subgroups, less than 0.1% of genotype calls were for private GWAS associated SNPs (Additional file 8).

Groups K4 and K1 were also the largest subgroups in the panel, 252 and 146 genotypes, respectively (Additional file 8), so it is possible that more effect alleles, i.e., the minor alleles of GWAS loci in population, were found in these subpopulations due to the greater power bestowed on these groups by their larger sample sizes, and that more effect alleles would have been identified in groups K2, K3, and K5 if these population subgroup sizes had been on par with K4 (Additional file 8). Future diversity panels should try to include more individuals in these clusters to answer this question. However, we can say from our results that the individuals in group K4, which consisted of a mix of morphology types from across Africa, represent a good source of exotic diversity from which to breed for drought tolerance and biomass (Additional file 1).

### Peaks by trait, time, treatment, and location

GWAS peaks were classified in several ways based on the combinations of traits, time-points, treatments, and locations in which they registered as significant (Table 2, Additional files 4 and 5). Peaks were first classified as deviation (DV) and/or non-deviation (NDV) peaks. DV peaks were those peaks that were identified for any of the four phenotypes measured as deviations from the control treatment, and represent true tolerance loci, whereas NDV peaks were those identified using the raw phenotype data for each treatment. NDV peaks may be tolerance loci when identified under drought conditions for reasons of stochasticity, or, more likely, they may have an effect on the phenotype that is not dependent on the environmental condition.

**Table 2:** Distribution of GWAS peaks

	# NDV	% NDV	# DV	%DV
TRAIT				
B65	181	84.98	107	50.23
PH	151	70.89	129	60.56
BWET	150	70.42	88	41.31
LAI	57	26.76	30	14.08
TREATMENT				
Pre-flower during recovery	164	77.00	71	33.33
control	120	56.34	NA	NA
Post-flower during stress	117	54.93	127	59.62
Post-flower no stress	50	23.47	2	0.94
Pre-flower during stress	38	17.84	6	2.82
LOCATION				
WREC	168	78.87	129	60.56
KARE	159	74.65	63	29.58

Number of GWAS peaks identified using GWAS phenotype inputs by trait (B65 = biomass at 65% moisture content, tons/acre, PH = plant height, m, BWET = total fresh biomass, tons/acre, LAI = leaf area index) by treatment (pre-flower during recovery = pre-flowering drought treatment, late season - irrigation, pre-flower during stress = pre-flowering drought treatment, early season – no irrigation, post-flower during stress = post-flowering drought treatment, late season – no irrigation, post-flower no stress = post-flowering drought treatment, early season – irrigation, control = all control treatments), by location (WREC = Westside Research and Extension, KARE = U.C. Kearney). # NDV = the number of non-deviation GWAS peaks identified, i.e., GWAS peaks identified using the raw phenotype data, % NDV = percent of all GWAS peaks identified using the raw phenotype data, # DV = number of GWAS peaks identified using the deviation phenotype data (DV for pre-flowering treatments = control data – pre-flowering drought data, DV for post flowering treatments = Control data - post-flowering drought data. There is no DV for control data, hence the NA for these cells in the table.) %DV = percent of all GWAS peaks identified using DV data. Note that the %DV plus %NDV is greater than 100% because some peaks belong to both DV and NDV groups

**Fig. 7:**
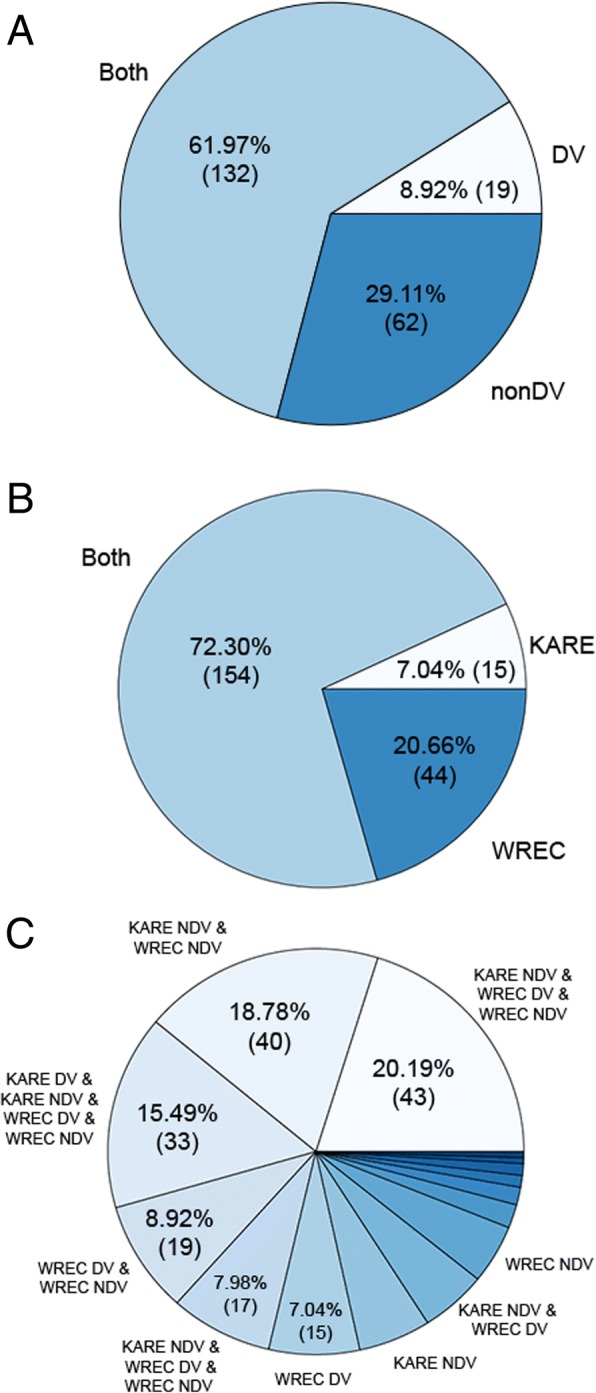
Distribution of GWAS peaks. Pie charts showing the proportion of GWAS peaks identified using deviation versus non-deviation phenotype data (**a**), at location = KARE (UC Kearney) versus location = WREC (Westside Research and Extension) (**b**)., and for all combinations of DV vs NDV at the two locations (**c**). **a** All peaks were classified as DV and/or NDV, where DV peaks were those identified using the phenotype data calculated as the deviation between either the pre-flowering stress treatment or the post-flowering stress treatment and the control, and the NDV peaks were those identified using the raw phenotype data. The majority of peaks were identified regardless of whether DV or NDV data were used. **c** Each wedge shows the proportion of the peaks that were only identified for a particular combination of data, e.g., the largest proportion of peaks (~ 20%) were identified only using NDV data from KARE, DV data from WREC and NDV data from WREC

Out of the final 213 GWAS peaks, ~ 29% (62) were exclusively NDV, ~ 9% [19] were exclusively DV, and ~ 62% (132) of the peaks were identified using both deviation and non-deviation data (Fig. 7a, Additional files 4 and 5). One strategy for deciding which peaks to begin validating first for drought tolerance would be to look at the peaks in either the DV or ‘both’ categories. The exclusively NDV peaks are an interesting source for future investigation, but are more likely to contain peaks linked to conversion than drought tolerance and so may be of lower priority for future validation studies. Similarly, more peaks were identified at both locations than at only one location (Fig. 7b, Additional files 4 and 5). Of the peaks identified at only a single location, more peaks were identified at WREC than at KARE, most likely as a result of the harsher drought stress conditions at KARE due to sandy loam soil conditions at this site. The classification of peaks based on the combination of both location and DV/NDV peak type is shown in Fig. 7c. Overall, the results suggest there is a high degree of overlap between peaks identified at different locations and using different phenotype data, which, in turn, suggests that the results, and in particular, the final set of filtered GWAS peaks, are of high quality and reliability.

**Fig. 8:**
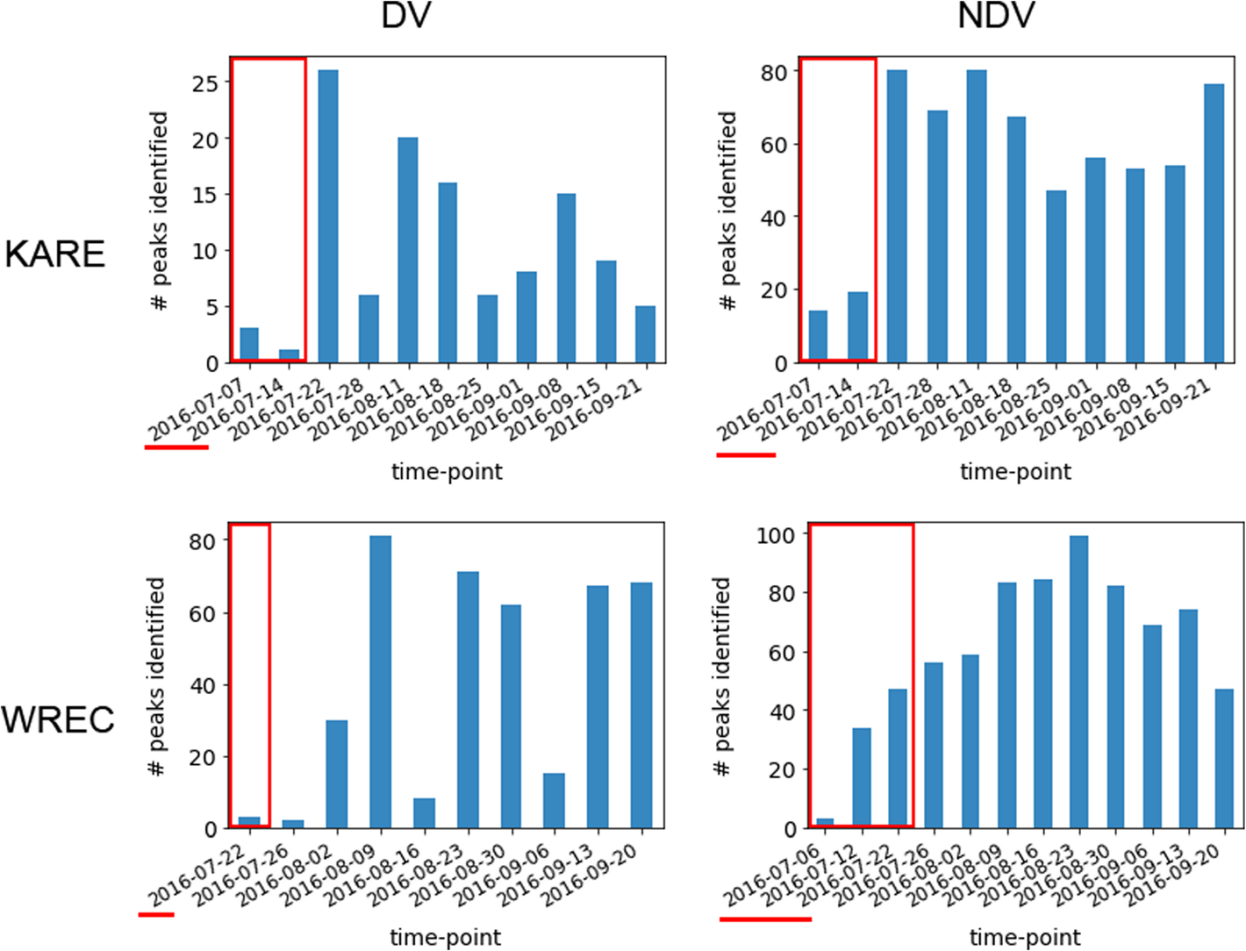
Distribution of peaks by date and location. Number of DV and NDV peaks identified at each location, by time-point. All peaks were classified as either DV, NDV, or as belonging to both groups, where DV peaks were those identified using the phenotype data calculated as the deviation between either the pre-flowering stress treatment or the post-flowering stress treatment and the control, and the NDV peaks were those identified using the raw phenotype data. Times surrounded by a red box and underlined with a red line are dates where the pre-flowering drought stress plots received no irrigation, and all other plots were irrigated. During the times not surrounded by the red boxes, the post-flowering drought stress plots received no irrigation, while all other plots were irrigated

For both the DV and NDV peaks, all four traits (i.e., B65, BWET, PH, and LAI) were on par with each other for the number of peaks they identified, with the exception of LAI, which was linked to noticeably fewer peaks than the other three phenotypes, likely as a result the higher error for this phenotype (Table 2, Additional files 4 and 5). The effect of treatment was examined in the context of time, where the majority of peaks were identified in pre-flowering treatments during recovery (i.e., late in the season after resumption of watering), control treatments, and post-flowering during stress time-points (i.e., late in the season during drought stress) (Table 2, Fig. 8, Additional files 4 and 5). Although these treatments also cover most of the time-points at which data were collected, the results suggest we identified alleles for both pre-flowering and post-flowering stress tolerance, with an enrichment for alleles active later in the growing season (Fig. 8).

### Candidate genes for biomass and drought tolerance

All annotated sorghum genes that fell into regions of each GWAS peak (as defined by the physical distance spanned by the significant SNPs for each peak) were pulled from PhytoMine to generate lists of candidate genes (Additional file 9). From these lists, 156 of the strongest candidate genes were selected as the entries closest to the peak GWAS SNPs with domains plausibly involved in drought tolerance and/or biomass accumulation (Additional file 10). Of these 156 candidates, ~ 48 were considered exceptionally strong candidates, yellow highlighted rows in Additional file 10, which include among other candidates, heat shock proteins, cytochromes, and antifreeze proteins, as well as proteins with repeat domains thought to be important for a variety of stress tolerance traits in plants such as Leucine rich repeats, Armadillo repeats, Ankyrin repeats, and WD40 repeats [29–31]. There was also a high prevalence of genes among the top candidates (~ 27) with DNA binding domains suggesting potential transcriptional regulators, as well as many genes (~ 21) that could otherwise be active in gene regulation, such as protein kinases and phosphatases [32] (Additional file 10).

The vast majority of the candidates were uncharacterized beyond protein domains and domain ontology or fell in what appear to be intergenic regions (166/213 or 78% of the peaks), which suggests there is a great potential to better understand plant genes that confer drought tolerance and increased biomass traits under drought conditions. Of the significantly enriched gene ontology (GO) terms among all genes falling into the regions spanned by the 213 significant GWAS peaks, the top 10 included domains essential to oxidoreductase activity and the treatment reactive oxygen species (ROS), which are both stress regulatory signals as well as destructive cellular compounds that must be neutralized under stressful conditions [33, 34]. Other significantly enriched GO terms include those involved in signal transduction and secondary messaging, functions that are highly likely to be involved in adaptive or acclimatizing processes [32] (Additional file 11).

The top 10 significantly enriched protein domains suggested potentially important roles for hydrolases, F-box transcription factors, proteins involved in the circadian cycle, and unknown protein domains (Additional file 12). Notably, hydrolases have been previously implicated in plant stress tolerance stress [35], and circadian proteins have clear ties to all forms of abiotic stress tolerance [36].

### Comparison to previous GWAS

With the exception of plant height, our traits have not been previously studied in sorghum. Due to the fact that our population consisted almost entirely of SC lines, the largest effects for both of these traits are the result of genetic differences between converted and non-converted lines in the panel, and variation between lines that had differing combinations of maturity alleles and dwarfing alleles. Thus, unsurprisingly, we identified large QTL in the regions of Ma1, Dw2, Dw3, and Dw1 in agreement with previously performed GWAS in sorghum [16, 21, 22, 27, 28, 37–39] (Additional file 10: green rows).

Few GWAS have been performed under drought conditions in sorghum. The largest was performed by Lasky et al. (2015), for harvest index plasticity compared between well-watered and drought treatments, relative net root growth as compared between control and Al toxicity treatments, and panicle weight plasticity compared between well-watered and drought treatments. Twenty eight of our GWAS peaks overlapped with peaks identified for one or more of these three traits and suggest strong candidates for further functional validation (Additional file 10).

A small number of our peaks also overlapped with sorghum plant architecture traits identified in a couple of other recent GWAS, and are summarized in Additional file 13. Overall, 44/213 peaks or ~ 21% of our regions overlapped with significant peaks from previously performed GWAS, while the majority of our peaks, 169/213 or 79%, were identified here for the first time.

## Conclusions

Using a drone to collect precise measurements for biomass traits over the course of the growing season for multiple locations and drought stress treatments allowed us to identify 213 unique genomic regions associated with biomass and/or drought tolerance in sorghum, 79% of which were identified for the first time, and 100% of which were identified multiple times within our high-density phenotype trials. On average, the number of similar peaks identified in other, recently performed GWAS using the Sorghum Association Panel (SAP) [40], and a variety of conventional (i.e., non-phenomics) phenotyping approaches for traits related to those focused on in this study, was ~ 15.3 associations per trait [21, 27, 37, 39, 41–43]. Using our filtered set 619 daylength-insensitive lines, by contrast, we identified ~ 111.6 associations per trait, 13.7 times the number of results from the most comparable recent studies in the literature (Additional file 13). These results evidence the extraordinary power that we gained by using high-density, precision based phenomics (Additional file 13).

The 213 regions and candidate genes we identified in this study provide strong targets for future experiments focused on gene validation, with the ultimate goal of identifying new alleles to deploy in the breeding/engineering of drought tolerant sorghum varieties. We conclude that HDP-GWAS will be a powerful tool for identifying all manner of genotype-trait associations in sorghum, and likely, in a wide variety of other crop species, as well.

## Methods

### Site descriptions, planting, irrigation and harvesting

648 lines including 620 SC lines, two other inbred lines and 26 sorghum hybrids provided by the USDA-ARS National Genetic Resources Program (GRIN) and Chromatin Inc., respectively, were selected to make up the diversity panel in order to assay the natural diversity of *sorghum bicolor*. The two field locations were the University of California Agriculture & Natural Resources (UC-ANR) Kearney Agricultural Research & Extension Center (KARE) in Parlier, CA and the UC-ANR Westside Research and Extension Center (WREC) in Five Points, CA. One hundred seeds of each converted line from the Sorghum Conversion program were requested from GRIN (https://www.ars-grin.gov) in the fall of 2014 for planting at KARE in the summer of 2015. Single row plantings, 6.1 m in length on .76 m beds, occurred on June 1, 2015 on a Hanford sandy loam soil and fully irrigated throughout the growing season. Fifty plants per plot were bagged and harvested for increase. In some cases, open pollinated plants were collected because of poor seed set under bags. Seed was then threshed, cleaned and stored in 10 °C cold storage units.

The 620 SC lines and 26 Chromatin breeding lines and hybrids were planted in two row plots (6.1 × .76 m), along with 6 (x two row) plots of two inbred lines with characterized drought tolerance: RTx430, a pre-flowering drought tolerant line, and BTx642, a post-flowering drought tolerant line, on June 1, 2016 at KARE and June 6, 2016 at WREC using a four-row Almaco research row planter [44, 45]. All lines were planted in blocks of plantings that were adjacent to each other, and separated into a non-water stressed (control) treatment, a pre-flowering period water stress treatment (pre-flowering/PRE), and post-flowering period water-stress treatment (post-flowering/POST).

The soil type at WREC is a Panoche clay loam and the soil at KARE is a Hanford sandy loam. At both sites, daily potential evapotranspiration (ET_o_) was determined using on-site weather stations that are part of the CA Irrigation Management Information System (CIMIS), with the weather stations located approximately 150 m from the field plot sites at both KARE and WREC [46]. With the exception of two post-emergence sprinkler irrigations applied soon after planting at the WREC site, all irrigations at both sites were applied using gated-pipe furrow irrigation. Amounts of water applied during each irrigation event at the two field sites differed due to the lower water infiltration rates at the KARE site, resulting in typical furrow irrigation amounts averaging 55 mm per irrigation event at KARE versus an average of 117 mm per irrigation event at WREC.

To supplement winter rainfall and prepare the field for planting, all plots at KARE received a 150 mm furrow irrigation prior to planting, and all plots at WREC received an 85 mm pre-planting furrow irrigation. These irrigations provided adequate soil moisture for seed germination at KARE, but at WREC it was determined that due to drying, windy conditions immediately prior to and at planting, sprinklers were needed to apply an additional 89 mm of water between 6/07 and 6/17, 2016 to ensure uniform germination and seedling establishment.

The pre-flowering water stress treatment was imposed by providing no irrigation during the period from seedling emergence until the 50% average flowering growth stage, with the first within-season irrigations for that treatment on 7/29 and 7/18 for WREC and KARE, respectively. Irrigations at both sites continued after those dates at timings and amounts that matched the control treatments. Final irrigations in the pre-flower and control treatments were applied on 9/09 and 9/06 for WREC and KARE, respectively. The post-flowering drought treatments were irrigated on the same dates and amounts as the control treatment from post-emergence until 50% flowering, after which irrigations were terminated, 7/28 and 7/19 for WREC and KARE, respectively.

Additional file 14 shows ET_o_ and irrigation water application amounts for these representative growth stages and irrigation treatments. Values for ET_o_ are typical of those for summer-planted sorghum in the semi-arid, relatively hot Mediterranean-type climate of the San Joaquin Valley in central CA. Measured rainfall was essentially zero, with > 2 mm rainfall, total, at both sites from the June 1 to September 30 growing period (Additional file 14).

### Genotyping, imputation, and population structure analysis

DNA was extracted from young leaf tissue using ThermoFisher plant DNAzol® reagent according to the manufacturer’s directions [47]. GBS library prep and sequencing was then performed by the University of Wisconsin using the ApeKI for restriction digest, according to standard protocols [48]. Raw GBS data were processed using the TASSEL5 GBSV2 pipeline and tags were aligned to the Phytozome v3.0 reference genome using Bowtie2 (−R 8, other parameters set to defaults, alignment rate = 79.5%). SNPs were called using the Tassel 5 discoverySNPCallerPluginV2 based on a Tassel 5 production pipeline kmer database containing all publicly available sorghum GBS data at the time of publication (that used ApeKI for restriction digest), ~ 4,163,211 donor kmers (min kmer count = 10) and 7904 donor *S. bicolor* taxa [49, 50].

Imputation was performed using FILLIN (TASSEL 5) using the same kmer database, using FILLIN defaults [51, 52]. Proportion of unimputed SNPs was equal to 0.50 for minor allele homozygotes, 0.74 for heterozygotes, and 0.34 for major allele homozygotes. Using masking of known alleles, imputation accuracy was found to be 93% for minor allele homozygotes, 99% for major allele homozygotes, and 50% for heterozygotes. For all 355,378 masked vs unmasked site comparisons, the R^2^ value was equal to 92%. Given that the overall rate of heterozygosity in the population was quite low these values were deemed acceptable for accurate GWAS analysis.

Post-imputation, all SNPs with call rates < 75% were removed from the dataset for a total of 272,942 SNPs. Two individuals (358 and 638) with call rates ≤60% were also removed from the dataset. All other individuals had call rates ≥60%. Prior to GWAS analysis, SNPs were also filtered for minor allele frequency (MAF) ≥0.01, for a final GWAS dataset of 131,544 SNPs. In this final SNP dataset, an average of ~ 76.81% of genotype calls across each taxa were homozygous for the major allele, ~ 12.88% were homozygous for the minor allele, and 1.77% were heterozygous. Neighbor joining trees were created in R using the function nj from the ape library [53]. PAMK was performed using function pamk from R package fpc [54], and PCA was performed using R function prcomp.

### Linkage disequilibrium calculation

Pairwise linkage disequilibrium (LD) was calculated for all SNPs on each chromosome using Plinkv1.9, function = −-r^2^ -inter-chr -yes-really. Using the resulting LD matrices, the relationship between SNP pairwise distance (BP_B - BP_A) and LD, for each chromosome, was calculated as the Gaussian smoothed r^2^ by SNP pairwise distance, where smoothed r^2^ was found using the Gaussian kernel from Python3 scipy, function = scipy.ndimage.filters.gaussian_filter (σ = 500). Local LD was then defined as the smoothed r^2^ that corresponded to the location of a given SNP in the resulting tables of BP_A, BP_B, pairwise distance, and smoothed r^2^.

### Phenotyping

The drone used to collect phenotyping data was a modified multirotor vehicle and Blue River’s proprietary imaging payload. The vehicle was a six propeller multirotor, flown at 25 m altitude and 6.5 m/s airspeed using an onboard autopilot with GPS waypoint navigation to ensure the target image resolution of 1 cm was achieved. The drone measured four plant traits: plant height (m, PH), leaf area index (LAI), fresh total plant fresh biomass (tons/acre, BWET), and vegetative biomass adjusted to 65% moisture content (tons/acre, B65). These trait measurements were derived from LiDAR (Light Detection and Ranging) and multispectral imagery collected by Blue River’s imaging payload. The LiDAR instrument used near-infrared lasers to create a 3D point cloud of the sorghum canopy < 3 cm spot spacing, which is in turn used to extract plant height. Images from the color and NIR cameras were stitched together into a single mosaic image of each field using the TraitMapper software. The color and near-infrared mosaics were then converted to reflectance, and a digital grid representing the field plots was overlaid onto the mosaics. The reflectance and LiDAR height data were then extracted for each field plot and converted to trait measurements using TraitMapper.

Ground-truthing measurements were taken over a small subset of field plots to calibrate and validate the drone-based measurements, which covered all field plots. Manual field-based measurements of PH, LAI, BWET, and B65 were taken at 15 plots per week (10 plots at KARE, 5 plots at WREC) within 1 day of each drone overflight. Due to the laborious nature of the field-based measurements, each of the 15 manual measurements were taken within a sub-plot (80 × 160 cm), centered within each field plot. The ground-truthing of PH was performed using a height pole to measure the height of all plants within each subplot and then the median height was reported for each subplot. Leaf area index (LAI) was ground-truthed by first measuring the surface area of every leaf within each subplot using a hand-held Licor LI-3000C portable leaf scanner, and then dividing the total leaf area by the area of the subplot. BWET was ground-truthed by harvesting and weighing the total plant mass of each subplot. B65 was ground-truthed by then drying harvested plant material for 5 days at 50 °C in forced air drying ovens and reweighing.

Performance of the drone-based models of height, leaf area, and biomass was assessed using the ground-truthing data and repeated k-fold cross validation (k = 5). Specifically, each of the models for PH, LAI, BWET, and B65 were first trained on a calibration dataset, randomly selected from 80% of the ground-truthing data, and the R^2^ was computed using the remaining 20% of the ground-truthing data. This process was then repeated 1000 times, each time randomly selecting a different 80% subset for calibration. Finally, the median R^2^ over the 1000 repetitions was computed and defined here as the “validation R^2^”. The validation R^2^ were: PH R^2^ = 0.98, *n* = 88; LAI R^2^ = 0.90, *n* = 63; BWET R^2^ = 0.90, *n* = 63; B65 R^2^ = 0.89, *n* = 100 (n is total number of ground-truthing measurements).

### Phenotype data filtering

For the drone-collected phenotype data, two plots for each treatment and each location (2 × 3 × 2), were mistakenly identified ‘74’ and ‘475’, respectively, and could not be disambiguated. As a result, all plots labeled either ‘74’ or ‘475’ were removed from the dataset prior to analysis.

Drone-collected phenotype data were also filtered for noise. For each single genotype x trait x treatment x location combination, the phenotype data were plotted over the 11 or 12 timepoints at which they were collected to ensure a biologically plausible growth pattern, (i.e., a plant should not double in height 1 week, then shrink back the following week). Time plots with sharp peaks or zig zags were interpreted as containing phenotype noise/outliers in need of filtering prior to performing GWAS. To standardize the removal of outliers and prevent the need to look at every time-plot by eye, a kernel smoothing spline line (σ = 2.5) was fit for each time plot. The standard deviation (sd) of the residuals was calculated and each plot was first classified as either high or low noise, where high noise plots had residual sd ≥0.4 for PH, ≥0.51 for LAI, ≥3.0 for B65, and ≥ 4.5 for BWET. Data points on high noise plots were considered outliers if the residual from the spline was ≥1.2 sd for PH, B65, and BWET, and ≥ 1.5 sd for LAI. Data points on low noise plots were considered outliers if the residual from the spline ≥2 sd for PH, B65, and BWET, and ≥ 3 sd for LAI. These parameters were determined empirically using 10–15 case (obvious outliers identified by eye) and 4–8 control plots (no outliers identified by eye) per trait, for a total set of 49 case and 25 control plots. Different sets of parameters were tested to select the set of final values that resulted in the removal of all conservatively defined outliers from the case plots and the removal no points from the control plots. Example of case and control sample plots are shown in Additional file 2: Figure S3.

### GWAS modeling

Individual single variate GWAS were run for each combination of trait, treatment, time-point and location using both the raw phenotype data, and the deviations of the drought treatments from the control, equal to the control phenotype – pre-flowering drought treatment phenotype and control phenotype – post-flowering drought phenotype for pre-flowering and post-flowering drought deviations, respectively, for a total of 460 individual GWAS runs. All GWAS were run using GEMMA [55]. IBD matrices were calculated for each GWAS model using GEMMA (−gk 2). To control for population structure and determine the best GWAS model for each phenotype datafile, ~ 9 different covariate files were tested for each model: no covariates, the first principle component (PC), the first two PCs, the first three PCs, the first four PCs, then, the first PC with flowering time data, the second two PCs with flowering time data, the first three PCs with flowering time data, and the first four PCs with flowering time data. Because the KARE and WREC flowering times were collected in different ways, i.e., KARE FT data was collected in the field by an experienced sorghum breeder and WREC FT data were determined from manual examination of drone photographs, for the WREC GWAS runs, each covariate file that contained flowering times was tested using 1. The flowering times from WREC, as well as 2. The flowering times from KARE.

For every GWAS model, a QQ-plot was generated, and the simplest model (meaning the model with the fewest covariates) resulting in the QQ-plot with the least deviation from the null hypothesis was selected as the final GWAS model for a given data-file. Multiple test correction was performed using Benjamini-Hochberg method to a false discovery rate (FDR) of 0.1 [56].

After all single-variate GWAS models were selected, all SNPs that passed the significance threshold after multiple test correction were concatenated into a single results file for processing using a custom HDP-GWAS pipeline (Fig. 3).

### Custom HDP-GWAS peak definition pipeline

Conserved and reliable GWAS peaks were defined using the following pipeline, illustrated in Fig. 3. The following is a detailed explanation of the peak definition pipeline discussed in the results section.

#### Calculate local LD around significant SNPs

For each significant SNP, the SNP position was found in the table of values containing the kernel smoothed r^2^ by physical position and bp pairwise distance. SNPs were considered linked to a given significant SNP the pairwise kernel smoothed r^2^ was ≥0.3 for chromosomes 6 and 9, and considered linked if r^2^ was ≥0.2 for all other chromosomes. The higher LD threshold was used for chromosomes 6 and 9 as a result for the higher baseline LD on these chromosomes. Max distance was defined as the largest distance spanned by linked SNPs around a given significant SNP, and calculated as maximum base pair (bp) distance between SNPs defined as linked based on the above local LD parameters (Fig. 3 steps 1–4).

#### Group significant SNPs into preliminary peaks based on calculated local LD

SNPs in the concatenated table of all significant GWAS SNPs were considered to be a part of the same GWAS peak if they fell within the max distance defined for a given significant SNP, as determined in step one of the pipeline (Fig. 3 steps 5–9).

#### Zoom in on each GWAS peak

‘Zoomed’ Manhattan plots were constructed for each preliminary peak as defined in step two by plotting all SNPs +/− 50 Kb around the boundaries of each preliminary GWAS peak, for each individual GWAS run in which a given peak was identified, e.g., if a peak was identified in 12/260 GWAS runs, then 12 zoomed Manhattan plots were drawn, each showing only the SNPs +/− 50 Kb around the boundaries of the defined GWAS peak. Then, for each preliminary peak, all zoomed Manhattan plots were studied individually to determine if, based on the pattern of the graphed points, a preliminary peak needed to be divided into two or more peaks. If it was determined that a peak should be split into two or more peaks, it was re-plotted for all individual GWAS runs in which the peak was identified including all SNPs +/− 2 Mb around the peak boundaries (zooming out), in order to confirm the diagnosis. In some cases, zooming back out around peaks reversed an initial decision to the split the peak. In this way, a final set of refined peaks was determined (Fig. 3 steps 10–12).

#### Rate GWAS peaks for reliability and drop low reliability peaks from final GWAS result set

After the refined set of peaks was defined as described in step 3, a final set of zoomed Manhattan plots were constructed that including all SNPs +/− 50 Kb around the boundaries of each GWAS peak for each individual run in which a given GWAS peak was identified. Each of these zoomed Manhattan plots was then individually assessed and rated as either 1, 2, or 3 based on how likely a peak was to be the result of a data artifact such as population structure. Plots were rated ‘1’ when they were considered highly likely to be the result of a data artifact, i.e., cases where only a single SNP was high above the significance threshold with no linked SNPs leading to it (artifact due to the nature of linkage), and cases where there were not enough SNPs in the peak region to a draw a strong conclusion. By contrast, peaks were rated ‘3’ when they had very clear evidence to suggest they were not the result of data artifact, i.e., peaks were many linked SNPs lead to multiple SNPs that passed the significance threshold. All cases/peaks that fell between these two extremes and were, in other words, difficult to categorize as either definitely artifacts, or definitely not artifacts, were rated as ‘2’. Peaks with only plots rated as 1 were dropped from the final list of GWAS peak results (Fig. 3 steps 14–16).

Manhattan blots and Manhattan blot B’s were created using matplotlib.pyplot.scatter, where point size was set equal the total number of GWAS hits for each SNP (or each peak for M-blot B) * 200.

### Allele effects and PVE calculations

Percent variance explained (PVE) was calculated for each SNP, for each GWAS, as the squared Pearson correlation between the Best Linear Unbiased Predictors (BLUPs) of the given GWAS phenotype vector and the given SNP genotype vector, * 100. BLUPs were calculated in R using package rrBLUP, function ms.solve (phenotype vector, K = A.mat (genotype matrix). PVE summaries including mean, median, minimum, and maximum values were found by calculating the respective descriptive statistics across all individual PVE values found for each SNP, for each GWAS. PVE should be considered as a rough guide to the impact of each SNP in this population rather than a truly quantitative measurement because it may overestimate PVE in some cases due to the large number of SNPs and presence of subpopulation structure. For Manhattan blots, a PVE medians were calculated for all SNPs shown on the Manhattan blot by calculating the statistic across the PVEs for the subset of GWAS at which a given SNP was significant. Then for all the SNPs including in a particular GWAS peak on the Manhattan blot, the SNP with the highest median PVE was chosen to have its PVE plotted on the blot as a red star.

Allele effects were calculated as the differences between the mean phenotypes of the individuals homozygous for the effect/minor allele – the mean phenotypes of the individuals homozygous for the non-effect/major allele for each significant SNP identified for a given trait/phenotype for a particular trait x treatment x time x location combination. Allele effect heat maps were generated using Python3 seaborn.heatmap (cmp = “RedYlBu_r”), where the plotted allele effects were averaged across all time-points and locations.

### Candidate gene identification

Candidate genes were identified using PhytoMine v.3.0 using the template gene query (https://phytozome.jgi.doe.gov/phytomine/template.do?name=Region_Gene&scope=all), organism = *Sorghum bicolor*, chromosome = peak chromosome, location start > position of the first SNP associated with a particular peak, and location end < position of the last SNP associated with a particular peak (Additional files 4 and 5). Columns were selected from the ‘manage columns’ menu.

## Additional files

Additional file 1: Information about the lines used in this study. (XLSX 30 kb)

Additional file 2: This file contains **Figures S1**-**S10**. (PDF 8610 kb)

Additional file 3: Summary of pairwise LD across the ten chromosomes of *S. bicolor* in our GWAS diversity panel. (XLSX 17 kb)

Additional file 4: Summary of GWAS peaks identified using original trait data. (XLSX 137 kb)

Additional file 5: Summary of GWAS peaks identified using deviations between control and treatment data. (XLSX 65 kb)

Additional file 6: Summary of percent variance explained across 460 GWAS runs. (XLSX 131 kb)

Additional file 7: Average SNP allele effects for each GWAS peak by trait and treatment. (XLSX 50 kb)

Additional file 8: Number private GWAS SNPs by genetic subgroup. (XLSX 11 kb)

Additional file 9: Tables showing all genes in the regions spanned by significant SNPs for each peak. Gene information was obtained from the Phytomine tool in Phytozome v3.0. (ZIP 8760 kb)

Additional file 10: Top candidate genes from PhytoMine and literature for 213 GWAS peaks. (XLSX 232 kb)

Additional file 11: Significantly enriched gene ontologies. (XLSX 12 kb)

Additional file 12: Significantly enriched protein domains. (XLSX 43 kb)

Additional file 13: Generalized comparison of recent *Sorghum bicolor* GWAS results. (XLSX 11 kb)

Additional file 14: Potential evapotranspiration (ETo) and Irrigation water applications by treatment and date ranges in grain sorghum drought response study at the UC Kearney and UC Westside REC sites in 2016. (XLSX 10 kb)

## Abbreviations

B65: Biomass at 65% moisture content
BWET: Total fresh weight biomass
DV: Deviation (difference between drought and control)
GBS: Genotype by sequence
GWAS: Genome wide association study
HDP: High-density phenotype
KARE: Kearney Agricultural Research and Extension Center
Kb: Kilobase
LAI: Leaf area index
LD: Linkage disequilibrium
M-blot: Manhattan blot
NDV: Non-deviation
PH: Plant height
PVE: Percent variance explained
QTL: Quantitative trait loci
SC: Sorghum conversion
SCP: US Sorghum Conversion Program
SNP: Single nucleotide polymorphism
WREC: West Side Research & Extension Center
