# Supplementary material for: Association mapping by aerial drone reveals 213 genetic associations for Sorghum bicolor biomass traits under drought
Source: BMC Genomics. 2018 Sep 17;19:679. doi: 10.1186/s12864-018-5055-5 (PMC6142696; doi:10.1186/s12864-018-5055-5)
Supplement: Supplementary file 2 — This file contains Figures S1-S10. (PDF 8610 kb) [file 12864_2018_5055_MOESM2_ESM.pdf]

Supplementary figures

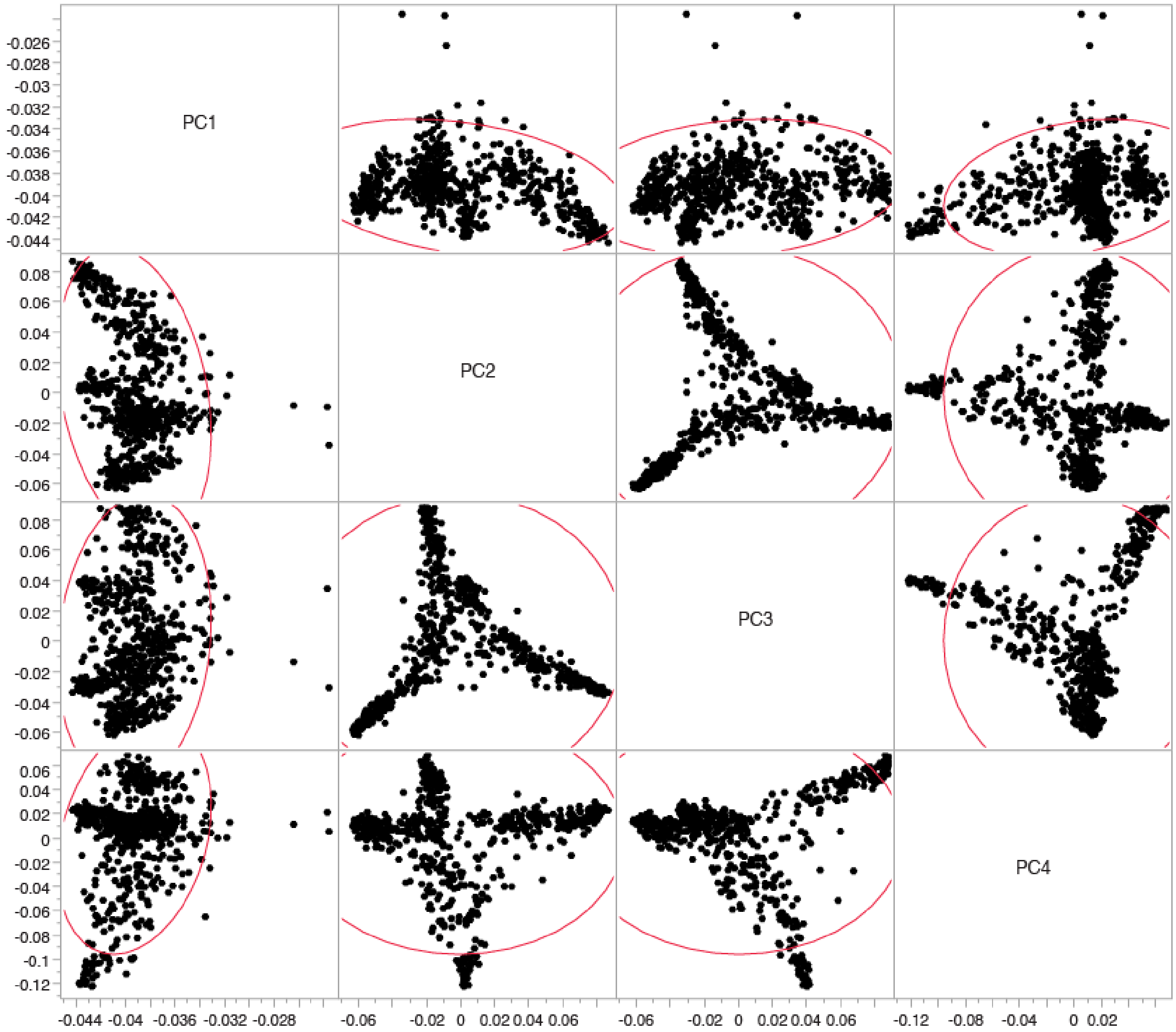

Figure S1. Scatterplot matrix of the first four principle components of the 646 *S. bicolor* GWAS diversity panel lines, calculated from the matrix of 183,989 bi-allelic SNPs with call rates ≥ 75%. Five subgroups are visible along the four axes of variation. Together, the first four PCs explain ~34% of the genotypic variance.

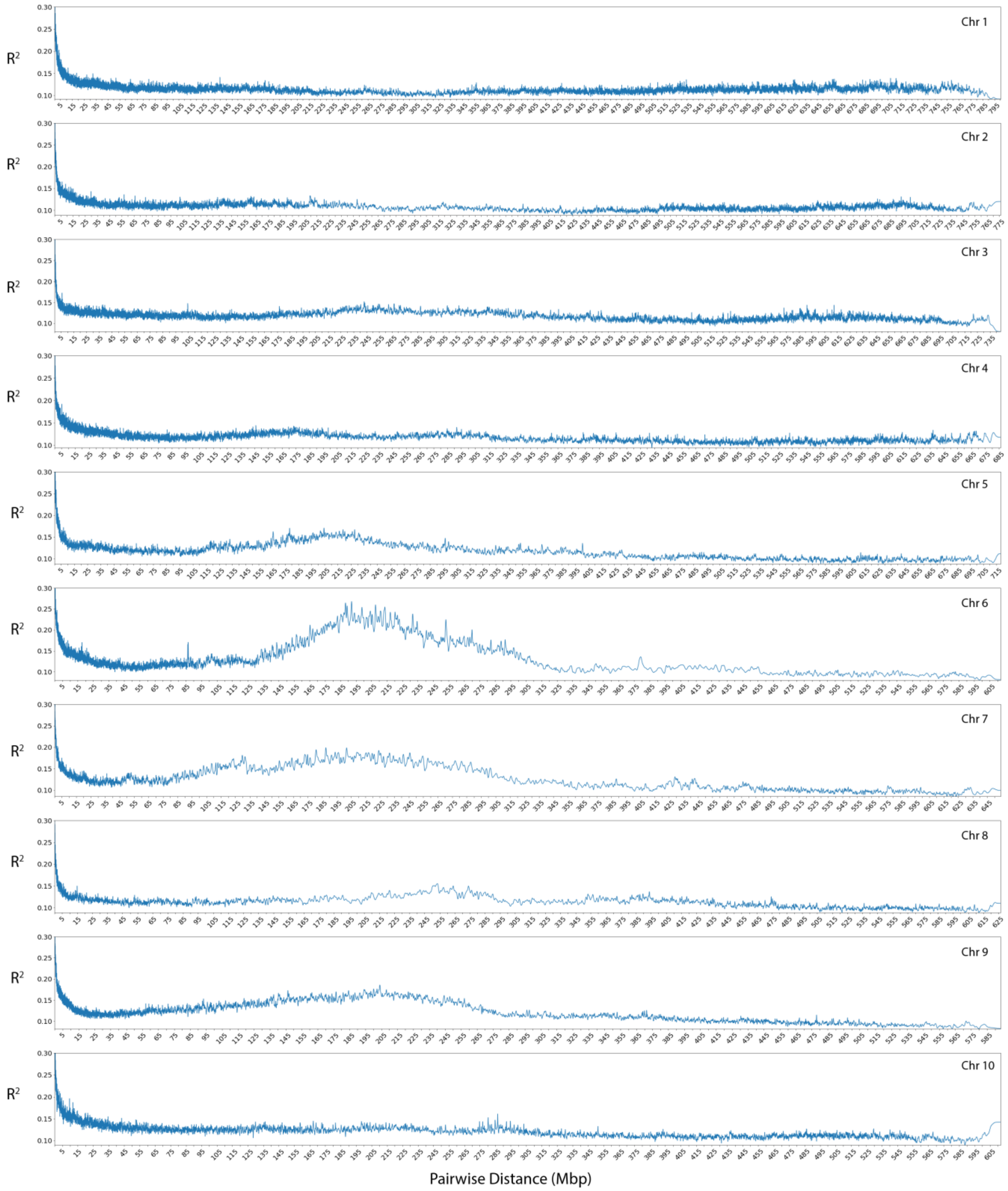

Figure S2. Gaussian kernel smoothed pairwise linkage disequilibrium (LD), r^2, by SNP pair distance (bp) for each chromosome. Pairwise LD was calculated for all pairs of SNPs on each chromosome using Plinkv1.9, and a Gaussian kernel smoother (σ = 500) fit to model the relationship between SNP distance and pairwise LD on each chromosome.

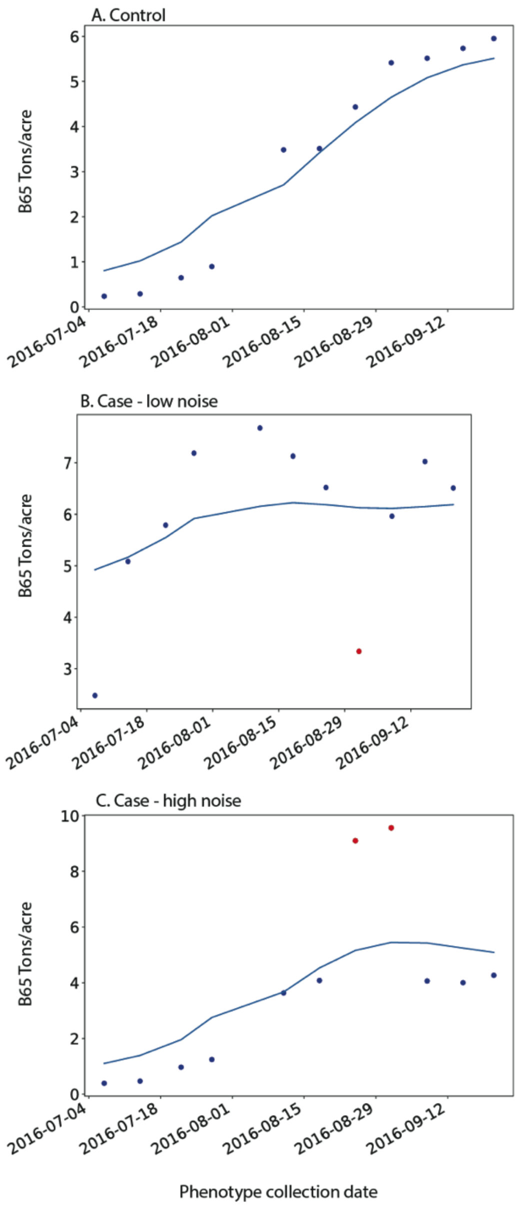

Figure S3. Illustration of phenotype outlier filtering process. For each individual, trait, treatment, and location combination the phenotype was plotted versus time and a kernel smoothing spline line fit ($\sigma = 2.5$) (blue line). The standard deviation (sd) of the residuals from the line were calculated and the plot was classified as high or low noise depending on the size of the sd. To tune the process of filtering outliers, a large number of plots were manually classified as either 'control', in which no points would be considered outliers(A) and 'cases' in which one or more points would be considered outliers - examples for a low noise case and high noise case are given in B and C, respectively. The points in red indicate outliers flagged and dropped by the chosen parameters after the tuning process. Plot A show raw B65 data at KARE-PRE-Plot 4, Plot B shows raw B65 data for KARE-control-Plot 174, and Plot C shows raw B65 data for KARE-PRE-Plot 116.

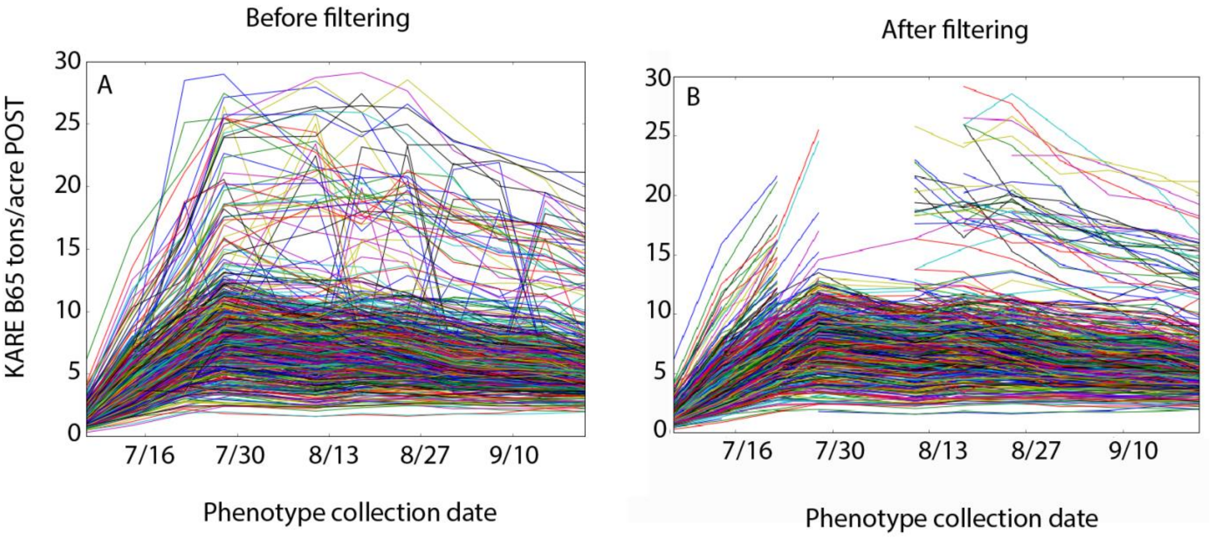

Figure S4. Example of B65 post-flowering phenotypes collected at KARE for all genotypes (a.k.a. plots), plotted over time, prior to any filtering of phenotype data (A), and after applying phenotype filtering (B). Prior to filtering, many phenotype outliers are present in the data, as evidenced by sharp increases and decreases of biomass over time on the line plots. After phenotype filtering and removal of outliers, the sharp increases and decreases are smoothed for a more biologically logical dataset.

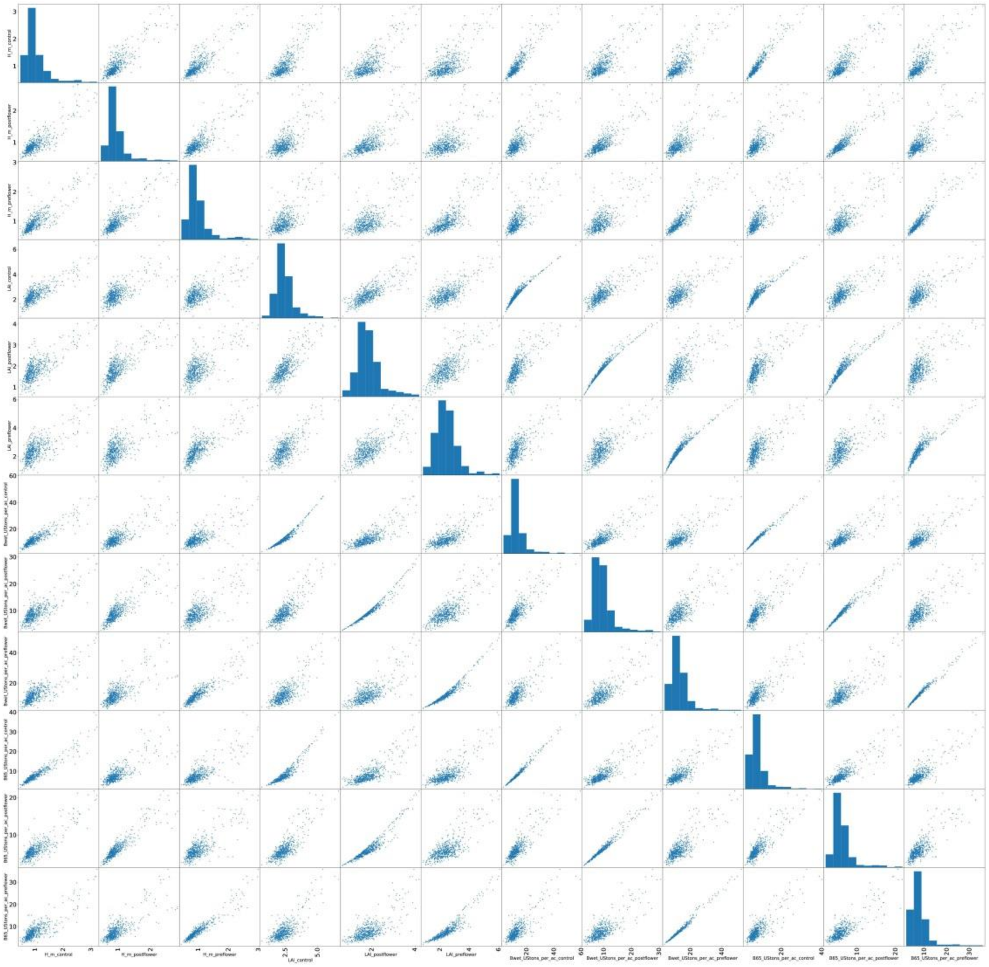

Figure S5. Example of end-of-season correlations for all phenotypes and treatments at KARE after phenotype outlier filtering. Correlations were generally positive between treatments and traits, as expected, and were generally consistent across time-points and locations. Central diagonal shows phenotype distributions.

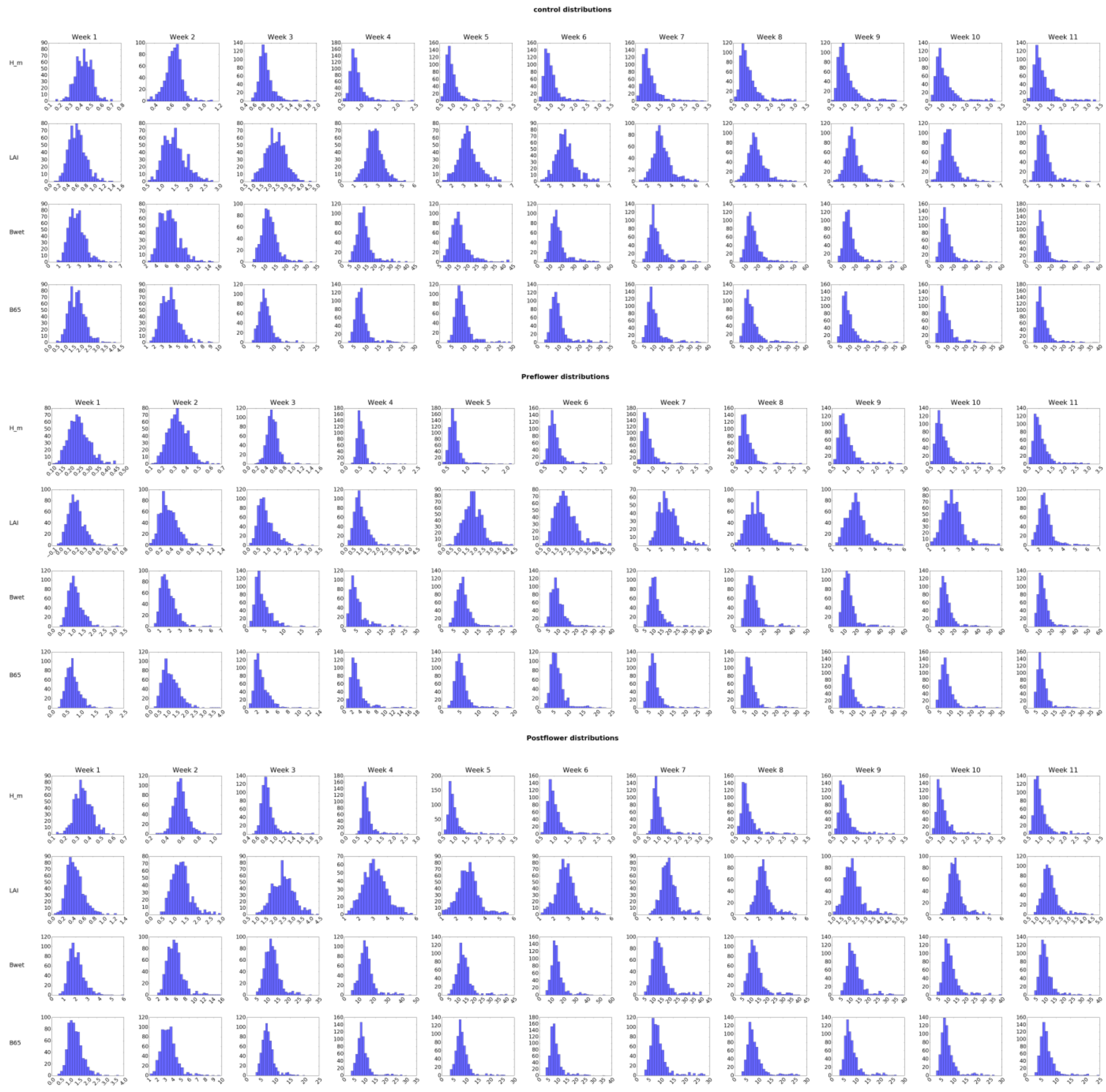

Figure S6. Distributions of drone collected phenotypes by week and by treatment and averaged by location.

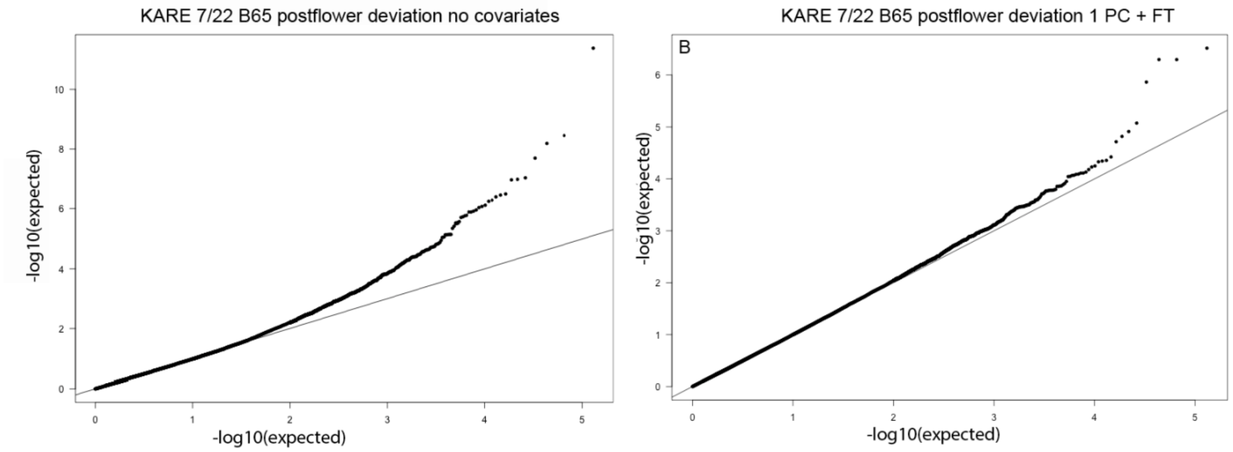

Figure S7. Examples of 2 QQ-plots for final 460 GWAS models. QQ plot on left (A) shows an example of a GWAS model that deviates significantly from the null hypothesis, most likely as a result of uncontrolled subpopulation structure, note that adding covariates to this particular GWAS model did not improve the QQ plot. QQ plot on right (B) shows a GWAS model with good fit, indicating the population structure is well controlled for this trait x time x treatment x location combination (TTTL).

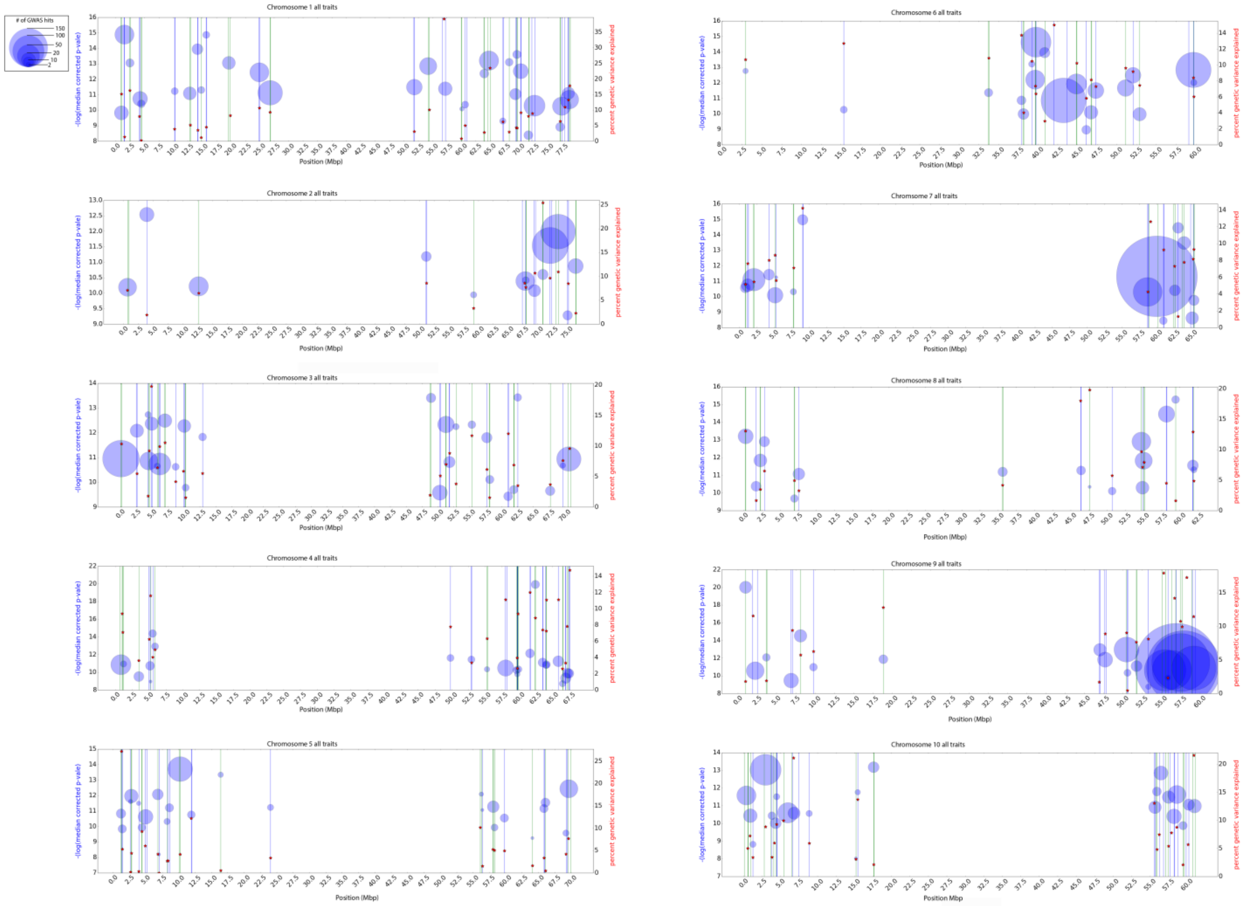

Figure S8. Manhattan blot Bs for chromosomes 1-10. The Manhattan blot B (M-blot B) is a variation on our Manhattan blot, a new method for viewing the results of a large number of single-variate GWAS, as might be performed to utilize HDP-GWAS data -- the results of 460 single-variate GWAS results in the case of the current study. As for the basic M-blot, each point on an M-blot B represents a SNP that was significant in at least one of the 460 GWAS, where the x-axis is the physical position of the SNP in Mb (by chromosome), and the left y-axis gives the median of the –log (FDR corrected p-value) across all GWAS where the SNP was significant. However, on the M-blot B, for each peak defined by our peak definition pipeline (the physical positions of the peaks are delineated using alternating blue and green lines), only the SNP with the highest median p-value across all significant SNPs that fell into the interval is shown, and the count of the number of times that SNP was identified in independent GWAS is summed to include its own counts as well as the sum of all counts of all other SNPs in the peak interval. The size of the point is then plotted as proportional to this grand sum for each peak. These M-blots show the combination of the results for all trait by treatment by time-point by location combinations. The right y-axis gives the value of the red star – the highest median percent variance explained (PVE) calculated for the SNPs within the interval of each peak, i.e., PVE was calculated for each SNP for each GWAS individually, then the median PVE was found for each SNP by taking the median PVE values across the GWAS where a given SNP was significant. The max PVE value per peak is the one plotted here.

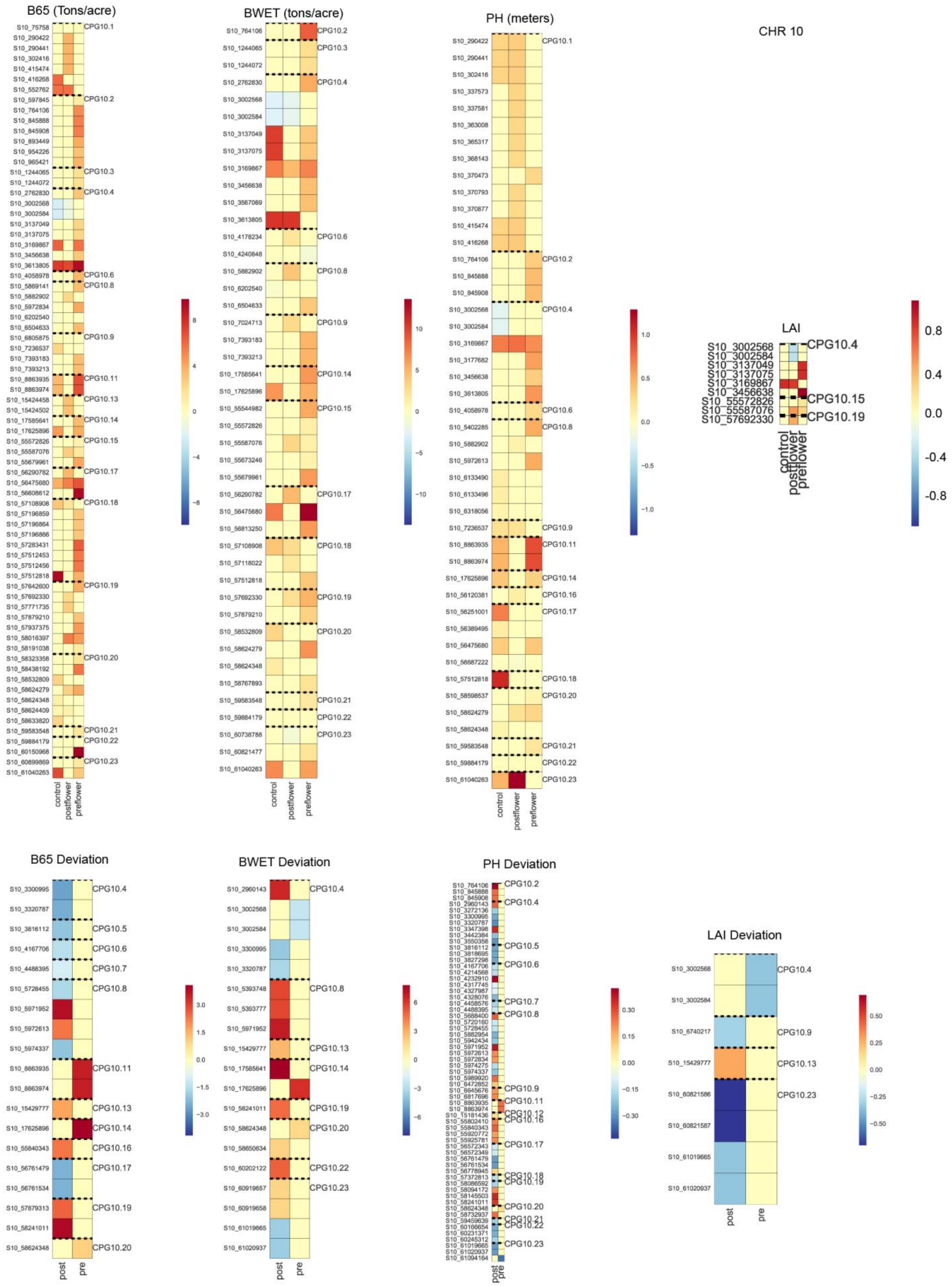
B65 (Tons/acre)
BWET (tons/acre)
PH (meters)
CHR 10
LAI
B65 Deviation
BWET Deviation
PH Deviation
LAI Deviation
control
postflower
preflower
post
pre

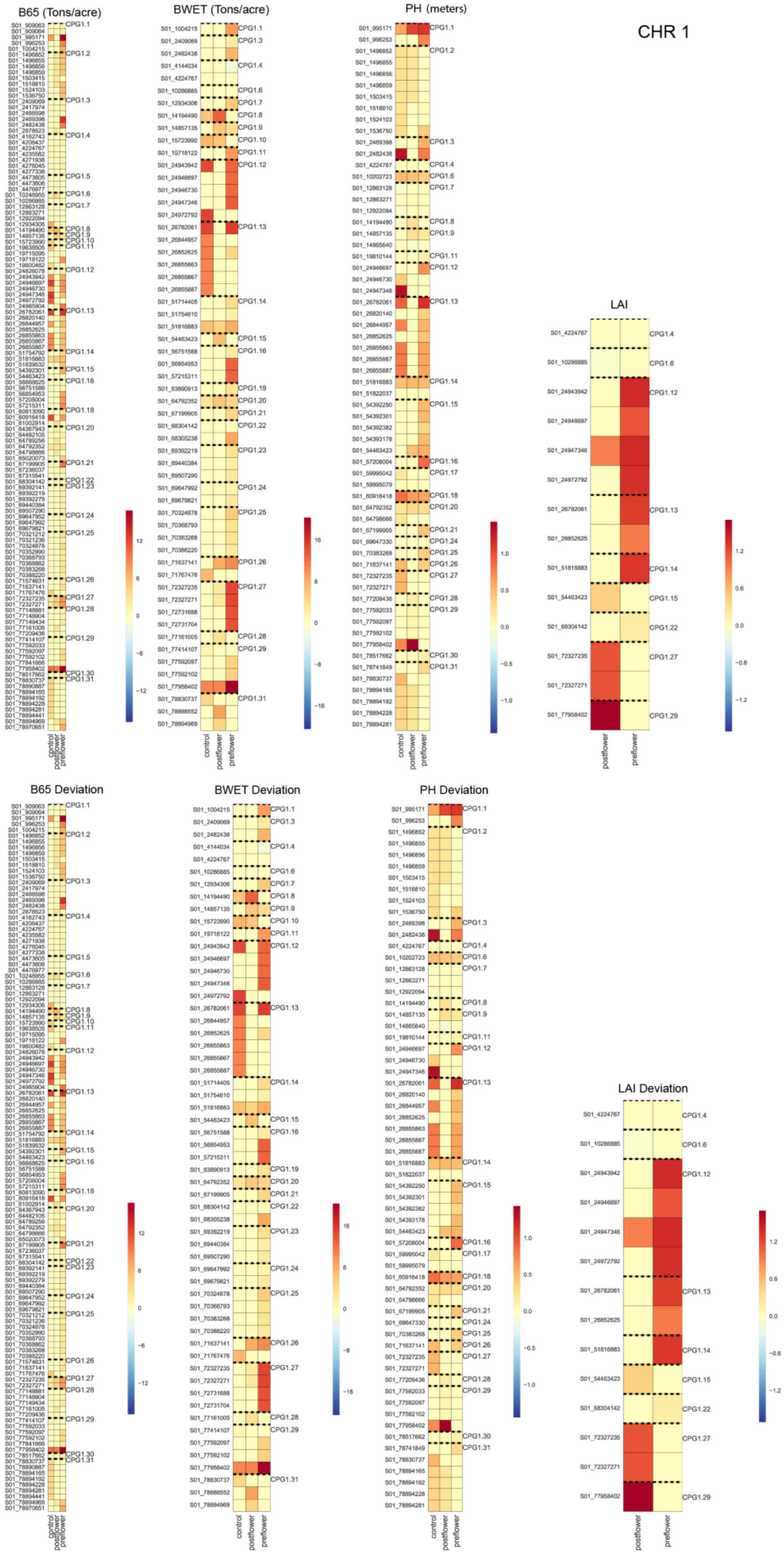

CHR 1
B65 (Tons/acre)
BWET (Tons/acre)
PH (meters)
LAI
B65 Deviation
BWET Deviation
PH Deviation
LAI Deviation
control
postflower
preflower

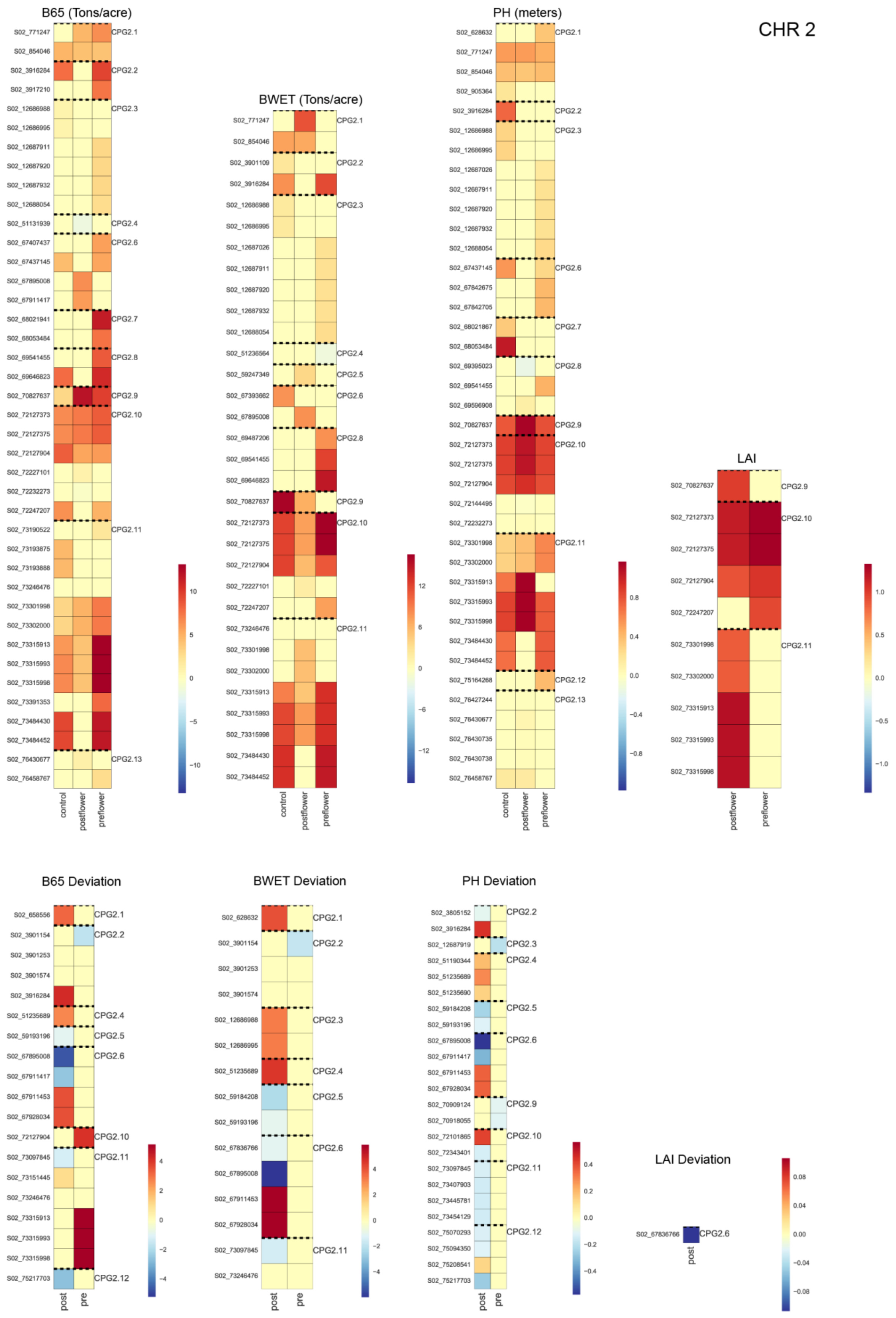
CHR 2
B65 (Tons/acre)
S02_771247 CPG2.1
S02_854046
S02_3916284 CPG2.2
S02_3917210
S02_12686988 CPG2.3
S02_12686995
S02_12687911
S02_12687920
S02_12687932
S02_12688054
S02_51131939 CPG2.4
S02_67407437 CPG2.6
S02_67437145
S02_67895008
S02_67911417
S02_68021941 CPG2.7
S02_68053484
S02_69541455 CPG2.8
S02_69646823
S02_70827637 CPG2.9
S02_72127373 CPG2.10
S02_72127375
S02_72127904
S02_72227101
S02_72232273
S02_72247207
S02_73190522 CPG2.11
S02_73193875
S02_73193888
S02_73246476
S02_73301998
S02_73302000
S02_73315913
S02_73315993
S02_73315998
S02_73391353
S02_73484430
S02_73484452
S02_76430677 CPG2.13
S02_76458767
control
postflower
preflower
10
5
0
−5
−10
BWET (Tons/acre)
S02_771247 CPG2.1
S02_854046
S02_3901109 CPG2.2
S02_3916284
S02_12686988 CPG2.3
S02_12686995
S02_12687026
S02_12687911
S02_12687920
S02_12687932
S02_12688054
S02_51236564 CPG2.4
S02_59247349 CPG2.5
S02_67393662 CPG2.6
S02_67895008
S02_69487206 CPG2.8
S02_69541455
S02_69646823
S02_70827637 CPG2.9
S02_72127373 CPG2.10
S02_72127375
S02_72127904
S02_72227101
S02_72247207
S02_73246476 CPG2.11
S02_73301998
S02_73302000
S02_73315913
S02_73315993
S02_73315998
S02_73484430
S02_73484452
control
postflower
preflower
12
6
0
−6
−12
PH (meters)
S02_628632 CPG2.1
S02_771247
S02_854046
S02_905364
S02_3916284 CPG2.2
S02_12686988 CPG2.3
S02_12686995
S02_12687026
S02_12687911
S02_12687920
S02_12687932
S02_12688054
S02_67437145 CPG2.6
S02_67842675
S02_67842705
S02_68021867 CPG2.7
S02_68053484
S02_69395023 CPG2.8
S02_69541455
S02_69596908
S02_70827637 CPG2.9
S02_72127373 CPG2.10
S02_72127375
S02_72127904
S02_72144495
S02_72232273
S02_73301998 CPG2.11
S02_73302000
S02_73315913
S02_73315993
S02_73315998
S02_73484430
S02_73484452
S02_75164268 CPG2.12
S02_76427244 CPG2.13
S02_76430677
S02_76430735
S02_76430738
S02_76458767
control
postflower
preflower
0.8
0.4
0.0
−0.4
−0.8
LAI
S02_70827637 CPG2.9
S02_72127373 CPG2.10
S02_72127375
S02_72127904
S02_72247207
S02_73301998 CPG2.11
S02_73302000
S02_73315913
S02_73315993
S02_73315998
postflower
preflower
1.0
0.5
0.0
−0.5
−1.0
B65 Deviation
S02_658556 CPG2.1
S02_3901154 CPG2.2
S02_3901253
S02_3901574
S02_3916284
S02_51235689 CPG2.4
S02_59193196 CPG2.5
S02_67895008 CPG2.6
S02_67911417
S02_67911453
S02_67928034
S02_72127904 CPG2.10
S02_73097845 CPG2.11
S02_73151445
S02_73246476
S02_73315913
S02_73315993
S02_73315998
S02_75217703 CPG2.12
post
pre
4
2
0
−2
−4
BWET Deviation
S02_628632 CPG2.1
S02_3901154 CPG2.2
S02_3901253
S02_3901574
S02_12686988 CPG2.3
S02_12686995
S02_51235689 CPG2.4
S02_59184208 CPG2.5
S02_59193196
S02_67836766 CPG2.6
S02_67895008
S02_67911453
S02_67928034
S02_73097845 CPG2.11
S02_73246476
post
pre
4
2
0
−2
−4
PH Deviation
S02_3805152 CPG2.2
S02_3916284
S02_12687919 CPG2.3
S02_51190344 CPG2.4
S02_51235689
S02_51235690
S02_59184208 CPG2.5
S02_59193196
S02_67895008 CPG2.6
S02_67911417
S02_67911453
S02_67928034
S02_70909124 CPG2.9
S02_70918055
S02_72101865 CPG2.10
S02_72343401
S02_73097845 CPG2.11
S02_73407903
S02_73445781
S02_73454129
S02_75070293 CPG2.12
S02_75094350
S02_75208541
S02_75217703
post
pre
0.4
0.2
0.0
−0.2
−0.4
LAI Deviation
S02_67836766 CPG2.6
post
0.08
0.04
0.00
−0.04
−0.08

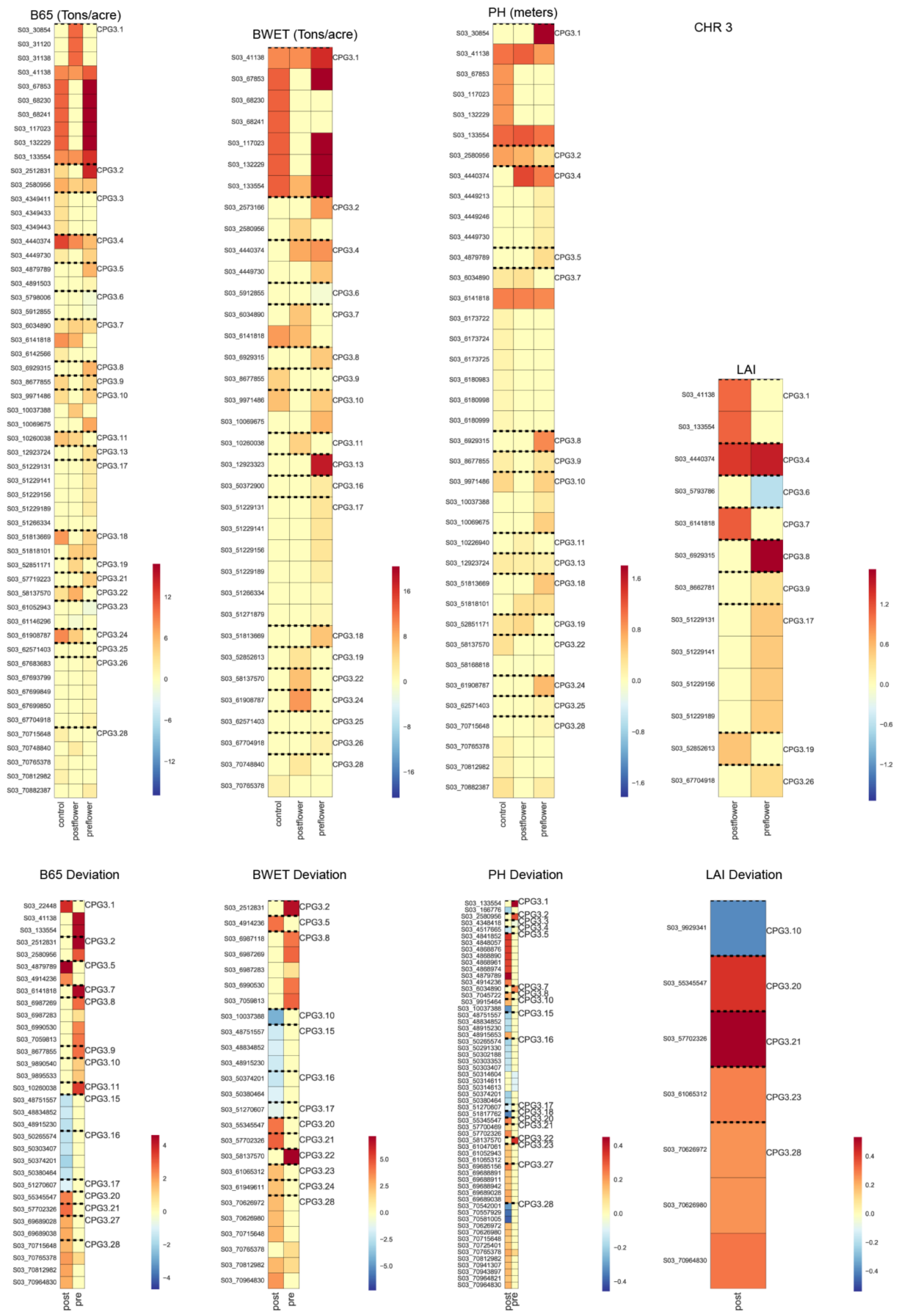

B65 (Tons/acre)
BWET (Tons/acre)
PH (meters)
CHR 3
LAI
control
postflower
preflower
B65 Deviation
BWET Deviation
PH Deviation
LAI Deviation
post
pre

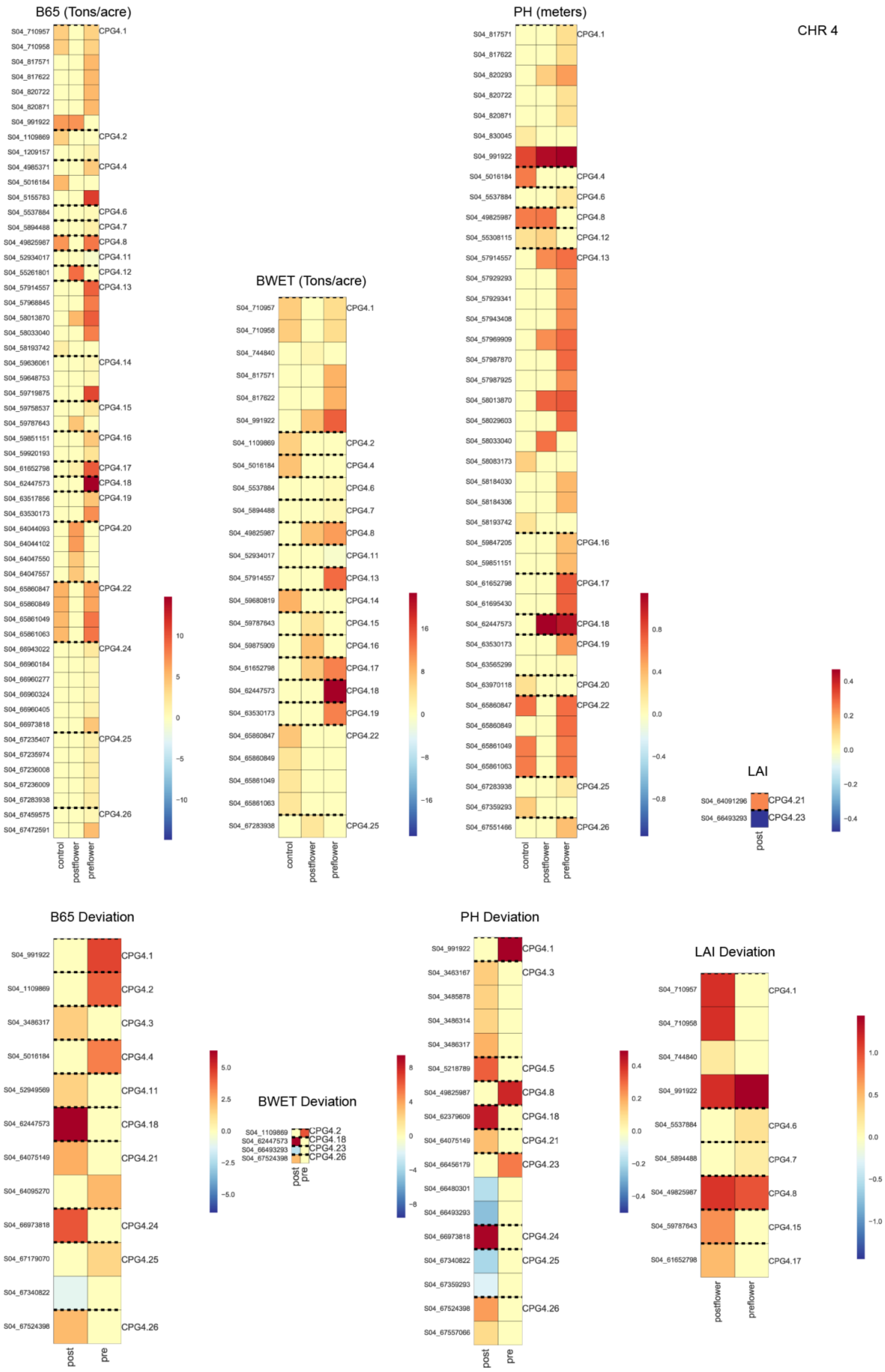

CHR 4
B65 (Tons/acre)
S04_710957
S04_710958
S04_817571
S04_817622
S04_820722
S04_820871
S04_991922
S04_1109869
S04_1209157
S04_4985371
S04_5016184
S04_5155783
S04_5537884
S04_5894488
S04_49825987
S04_52934017
S04_55261801
S04_57914557
S04_57968845
S04_58013870
S04_58033040
S04_58193742
S04_59636061
S04_59648753
S04_59719875
S04_59758537
S04_59787643
S04_59851151
S04_59920193
S04_61652798
S04_62447573
S04_63517856
S04_63530173
S04_64044093
S04_64044102
S04_64047550
S04_64047557
S04_65860847
S04_65860849
S04_65861049
S04_65861063
S04_66943022
S04_66960184
S04_66960277
S04_66960324
S04_66960405
S04_66973818
S04_67235407
S04_67235974
S04_67236008
S04_67236009
S04_67283938
S04_67459575
S04_67472591
CPG4.1
CPG4.2
CPG4.4
CPG4.6
CPG4.7
CPG4.8
CPG4.11
CPG4.12
CPG4.13
CPG4.14
CPG4.15
CPG4.16
CPG4.17
CPG4.18
CPG4.19
CPG4.20
CPG4.22
CPG4.24
CPG4.25
CPG4.26
control
postflower
preflower
10
5
0
−5
−10
BWET (Tons/acre)
S04_710957
S04_710958
S04_744840
S04_817571
S04_817622
S04_991922
S04_1109869
S04_5016184
S04_5537884
S04_5894488
S04_49825987
S04_52934017
S04_57914557
S04_59680819
S04_59787643
S04_59875909
S04_61652798
S04_62447573
S04_63530173
S04_65860847
S04_65860849
S04_65861049
S04_65861063
S04_67283938
CPG4.1
CPG4.2
CPG4.4
CPG4.6
CPG4.7
CPG4.8
CPG4.11
CPG4.13
CPG4.14
CPG4.15
CPG4.16
CPG4.17
CPG4.18
CPG4.19
CPG4.22
CPG4.25
control
postflower
preflower
16
8
0
−8
−16
PH (meters)
S04_817571
S04_817622
S04_820293
S04_820722
S04_820871
S04_830045
S04_991922
S04_5016184
S04_5537884
S04_49825987
S04_55308115
S04_57914557
S04_57929293
S04_57929341
S04_57943408
S04_57969909
S04_57987870
S04_57987925
S04_58013870
S04_58029603
S04_58033040
S04_58083173
S04_58184030
S04_58184306
S04_58193742
S04_59847205
S04_59851151
S04_61652798
S04_61695430
S04_62447573
S04_63530173
S04_63565299
S04_63970118
S04_65860847
S04_65860849
S04_65861049
S04_65861063
S04_67283938
S04_67359293
S04_67551466
CPG4.1
CPG4.4
CPG4.6
CPG4.8
CPG4.12
CPG4.13
CPG4.16
CPG4.17
CPG4.18
CPG4.19
CPG4.20
CPG4.22
CPG4.25
CPG4.26
control
postflower
preflower
0.8
0.4
0.0
−0.4
−0.8
LAI
S04_64091296
S04_66493293
CPG4.21
CPG4.23
post
0.4
0.2
0.0
−0.2
−0.4
B65 Deviation
S04_991922
S04_1109869
S04_3486317
S04_5016184
S04_52949569
S04_62447573
S04_64075149
S04_64095270
S04_66973818
S04_67179070
S04_67340822
S04_67524398
CPG4.1
CPG4.2
CPG4.3
CPG4.4
CPG4.11
CPG4.18
CPG4.21
CPG4.24
CPG4.25
CPG4.26
post
pre
5.0
2.5
0.0
−2.5
−5.0
BWET Deviation
S04_1109869
S04_62447573
S04_66493293
S04_67524398
CPG4.2
CPG4.18
CPG4.23
CPG4.26
post
pre
PH Deviation
S04_991922
S04_3463167
S04_3485878
S04_3486314
S04_3486317
S04_5218789
S04_49825987
S04_62379609
S04_64075149
S04_66456179
S04_66480301
S04_66493293
S04_66973818
S04_67340822
S04_67359293
S04_67524398
S04_67557066
CPG4.1
CPG4.3
CPG4.5
CPG4.8
CPG4.18
CPG4.21
CPG4.23
CPG4.24
CPG4.25
CPG4.26
post
pre
8
4
0
−4
−8
0.4
0.2
0.0
−0.2
−0.4
LAI Deviation
S04_710957
S04_710958
S04_744840
S04_991922
S04_5537884
S04_5894488
S04_49825987
S04_59787643
S04_61652798
CPG4.1
CPG4.6
CPG4.7
CPG4.8
CPG4.15
CPG4.17
postflower
preflower
1.0
0.5
0.0
−0.5
−1.0

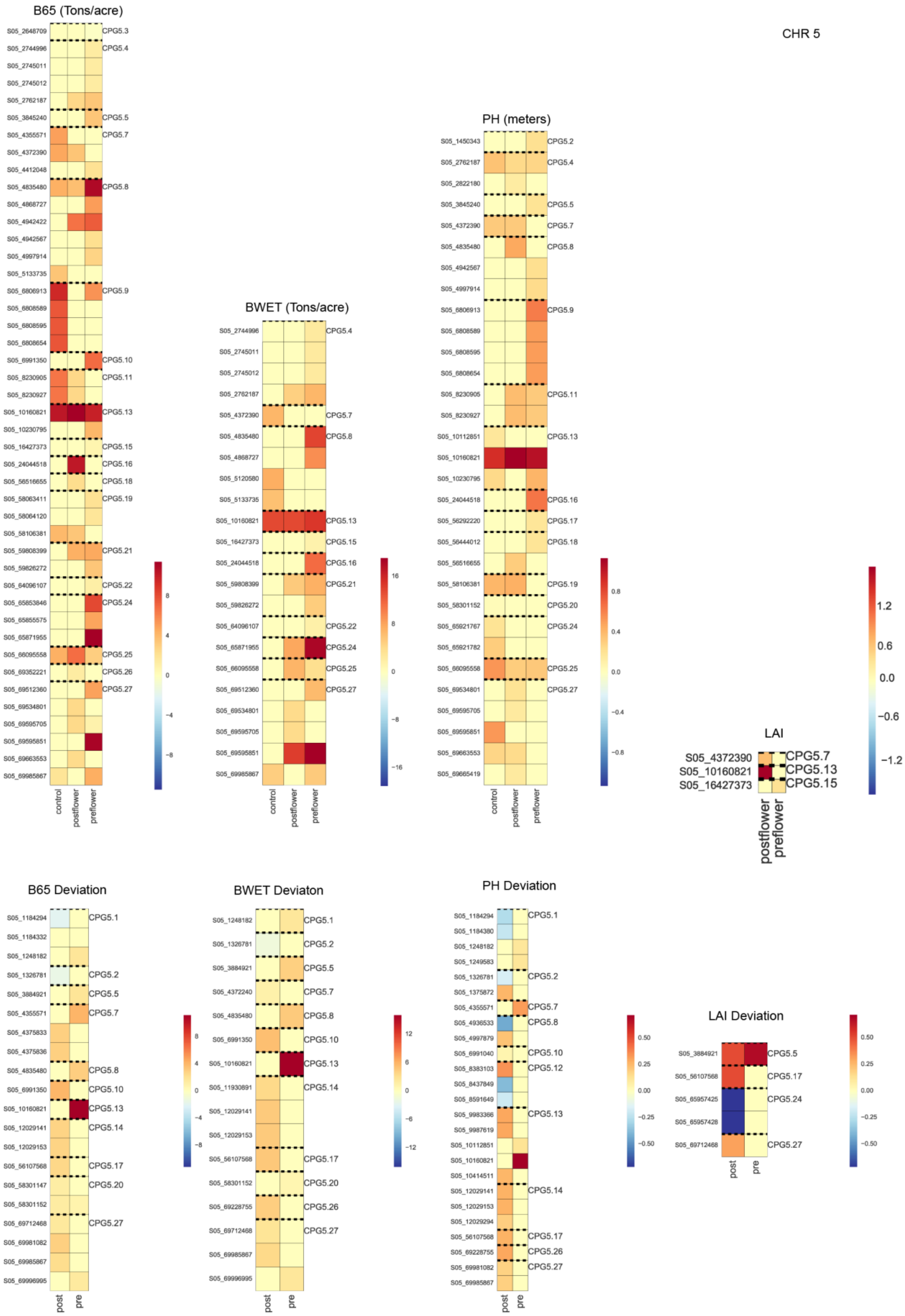
CHR 5
B65 (Tons/acre)
S05_2648709
S05_2744996
S05_2745011
S05_2745012
S05_2762187
S05_3845240
S05_4355571
S05_4372390
S05_4412048
S05_4835480
S05_4868727
S05_4942422
S05_4942567
S05_4997914
S05_5133735
S05_6806913
S05_6808589
S05_6808595
S05_6808654
S05_6991350
S05_8230905
S05_8230927
S05_10160821
S05_10230795
S05_16427373
S05_24044518
S05_56516655
S05_58063411
S05_58064120
S05_58106381
S05_59808399
S05_59826272
S05_64096107
S05_65853846
S05_65855575
S05_65871955
S05_66095558
S05_69352221
S05_69512360
S05_69534801
S05_69595705
S05_69595851
S05_69663553
S05_69985867
CPG5.3
CPG5.4
CPG5.5
CPG5.7
CPG5.8
CPG5.9
CPG5.10
CPG5.11
CPG5.13
CPG5.15
CPG5.16
CPG5.18
CPG5.19
CPG5.21
CPG5.22
CPG5.24
CPG5.25
CPG5.26
CPG5.27
control
postflower
preflower
8
4
0
−4
−8
BWET (Tons/acre)
S05_2744996
S05_2745011
S05_2745012
S05_2762187
S05_4372390
S05_4835480
S05_4868727
S05_5120580
S05_5133735
S05_10160821
S05_16427373
S05_24044518
S05_59808399
S05_59826272
S05_64096107
S05_65871955
S05_66095558
S05_69512360
S05_69534801
S05_69595705
S05_69595851
S05_69985867
CPG5.4
CPG5.7
CPG5.8
CPG5.13
CPG5.15
CPG5.16
CPG5.21
CPG5.22
CPG5.24
CPG5.25
CPG5.27
control
postflower
preflower
16
8
0
−8
−16
PH (meters)
S05_1450343
S05_2762187
S05_2822180
S05_3845240
S05_4372390
S05_4835480
S05_4942567
S05_4997914
S05_6806913
S05_6808589
S05_6808595
S05_6808654
S05_8230905
S05_8230927
S05_10112851
S05_10160821
S05_10230795
S05_24044518
S05_56292220
S05_56444012
S05_56516655
S05_58106381
S05_58301152
S05_65921767
S05_65921782
S05_66095558
S05_69534801
S05_69595705
S05_69595851
S05_69663553
S05_69665419
CPG5.2
CPG5.4
CPG5.5
CPG5.7
CPG5.8
CPG5.9
CPG5.11
CPG5.13
CPG5.16
CPG5.17
CPG5.18
CPG5.19
CPG5.20
CPG5.24
CPG5.25
CPG5.27
control
postflower
preflower
0.8
0.4
0.0
−0.4
−0.8
LAI
S05_4372390
S05_10160821
S05_16427373
CPG5.7
CPG5.13
CPG5.15
postflower
preflower
1.2
0.6
0.0
−0.6
−1.2
B65 Deviation
S05_1184294
S05_1184332
S05_1248182
S05_1326781
S05_3884921
S05_4355571
S05_4375833
S05_4375836
S05_4835480
S05_6991350
S05_10160821
S05_12029141
S05_12029153
S05_56107568
S05_58301147
S05_58301152
S05_69712468
S05_69981082
S05_69985867
S05_69996995
CPG5.1
CPG5.2
CPG5.5
CPG5.7
CPG5.8
CPG5.10
CPG5.13
CPG5.14
CPG5.17
CPG5.20
CPG5.27
post
pre
8
4
0
−4
−8
BWET Deviaton
S05_1248182
S05_1326781
S05_3884921
S05_4372240
S05_4835480
S05_6991350
S05_10160821
S05_11930891
S05_12029141
S05_12029153
S05_56107568
S05_58301152
S05_69228755
S05_69712468
S05_69985867
S05_69996995
CPG5.1
CPG5.2
CPG5.5
CPG5.7
CPG5.8
CPG5.10
CPG5.13
CPG5.14
CPG5.17
CPG5.20
CPG5.26
CPG5.27
post
pre
12
6
0
−6
−12
PH Deviation
S05_1184294
S05_1184380
S05_1248182
S05_1249583
S05_1326781
S05_1375872
S05_4355571
S05_4936533
S05_4997879
S05_6991040
S05_8383103
S05_8437849
S05_8591649
S05_9983366
S05_9987619
S05_10112851
S05_10160821
S05_10414511
S05_12029141
S05_12029153
S05_12029294
S05_56107568
S05_69228755
S05_69981082
S05_69985867
CPG5.1
CPG5.2
CPG5.7
CPG5.8
CPG5.10
CPG5.12
CPG5.13
CPG5.14
CPG5.17
CPG5.26
CPG5.27
post
pre
0.50
0.25
0.00
−0.25
−0.50
LAI Deviation
S05_3884921
S05_56107568
S05_65957425
S05_65957428
S05_69712468
CPG5.5
CPG5.17
CPG5.24
CPG5.27
post
pre
0.50
0.25
0.00
−0.25
−0.50

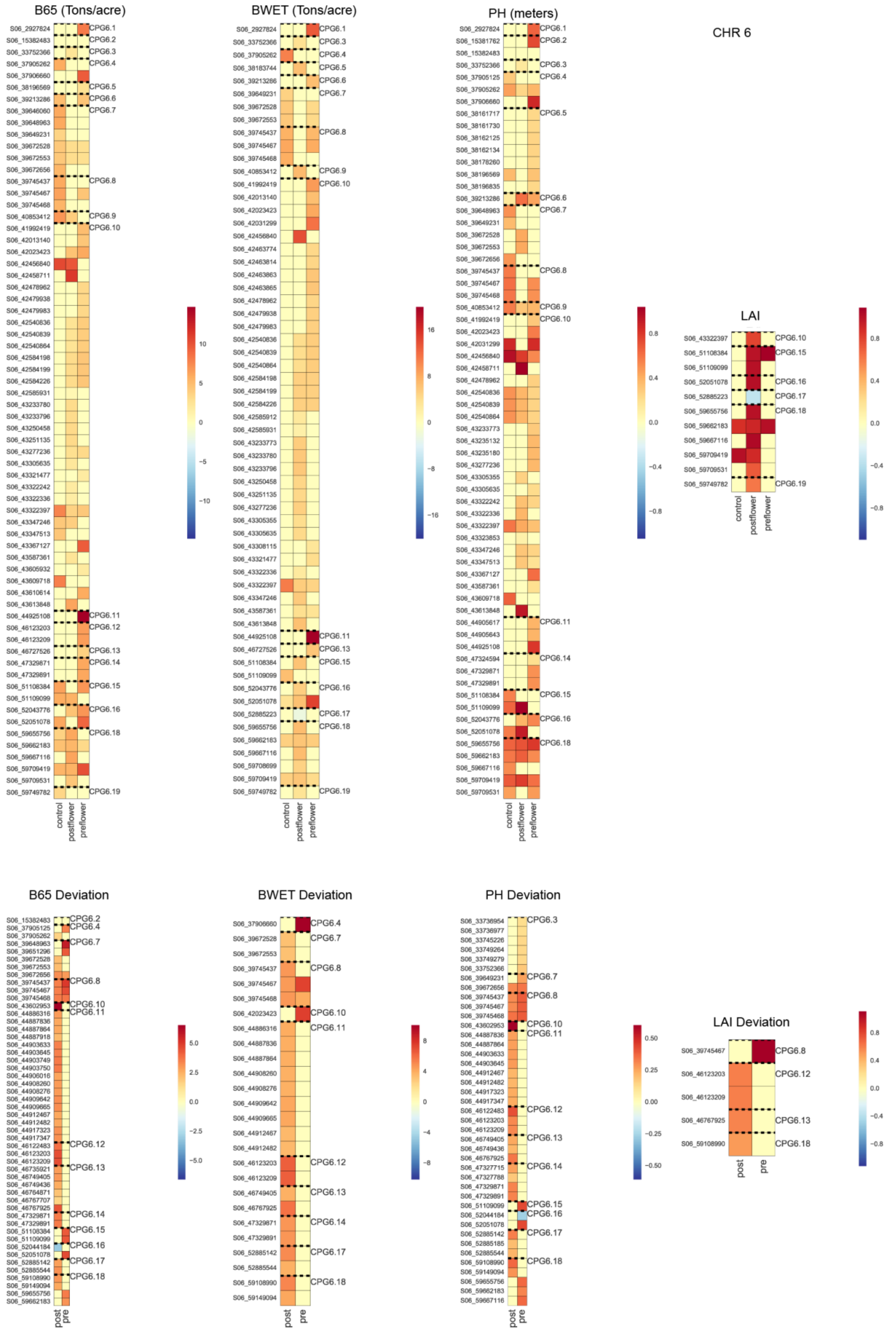

CHR 6
B65 (Tons/acre)
BWET (Tons/acre)
PH (meters)
LAI
control
postflower
preflower
B65 Deviation
BWET Deviation
PH Deviation
LAI Deviation
post
pre

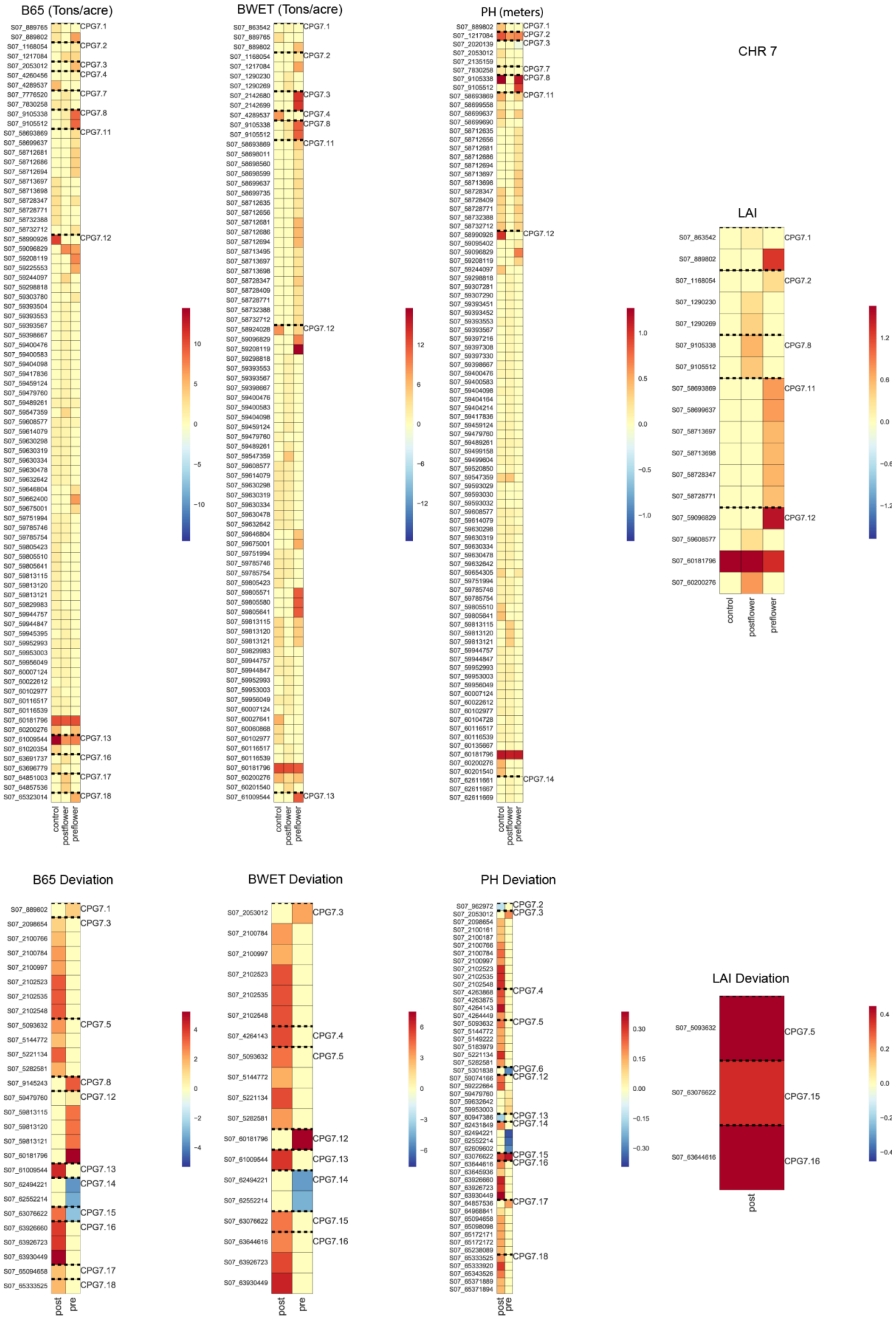

CHR 7
B65 (Tons/acre)
BWET (Tons/acre)
PH (meters)
LAI
control
postflower
preflower
B65 Deviation
BWET Deviation
PH Deviation
LAI Deviation
post
pre
CPG7.1
CPG7.2
CPG7.3
CPG7.4
CPG7.5
CPG7.6
CPG7.7
CPG7.8
CPG7.11
CPG7.12
CPG7.13
CPG7.14
CPG7.15
CPG7.16
CPG7.17
CPG7.18

CHR 8

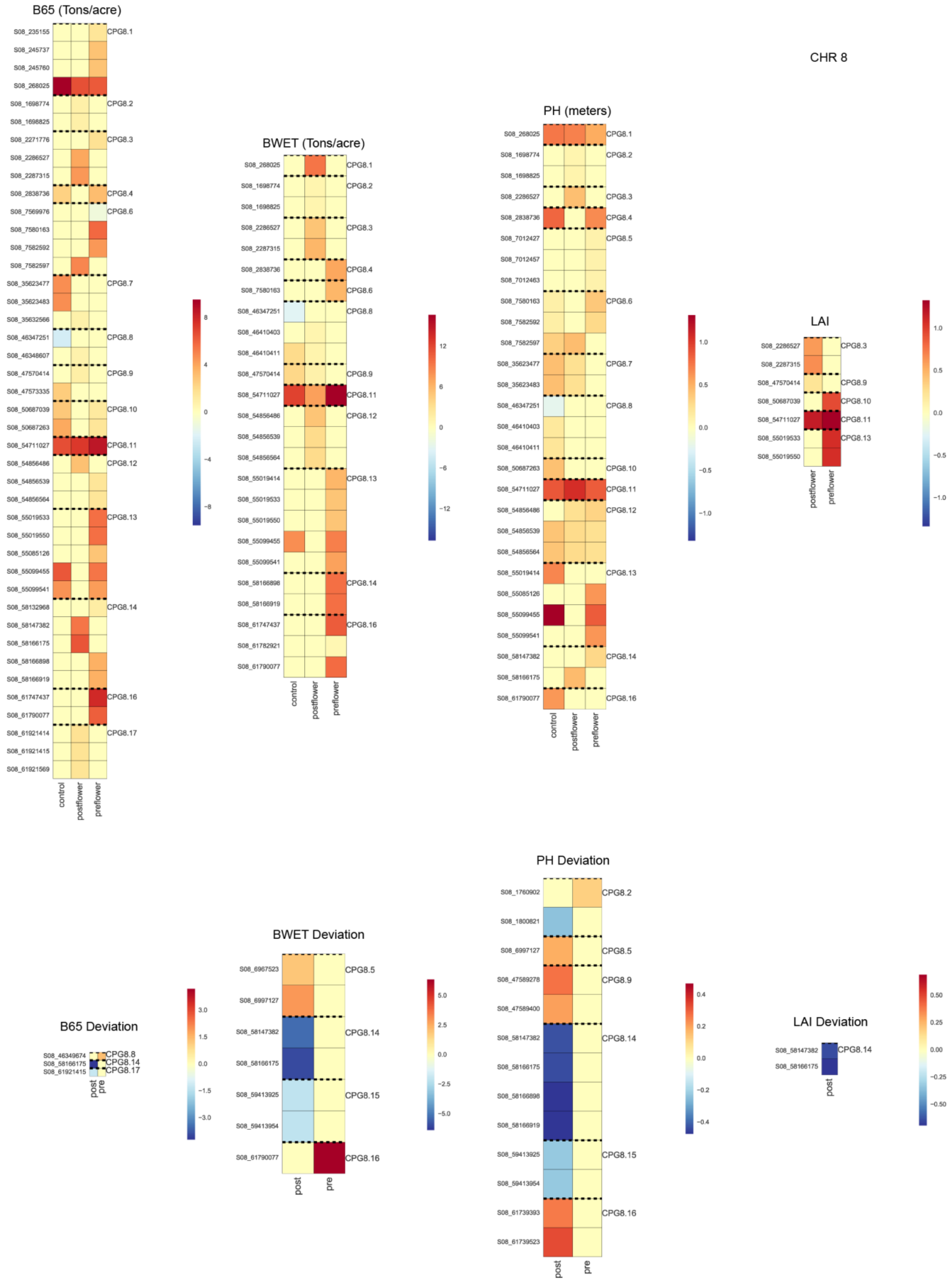
B65 (Tons/acre)
BWET (Tons/acre)
PH (meters)
LAI
control
postflower
preflower
CPG8.1
CPG8.2
CPG8.3
CPG8.4
CPG8.5
CPG8.6
CPG8.7
CPG8.8
CPG8.9
CPG8.10
CPG8.11
CPG8.12
CPG8.13
CPG8.14
CPG8.15
CPG8.16
CPG8.17
B65 Deviation
BWET Deviation
PH Deviation
LAI Deviation
post
pre

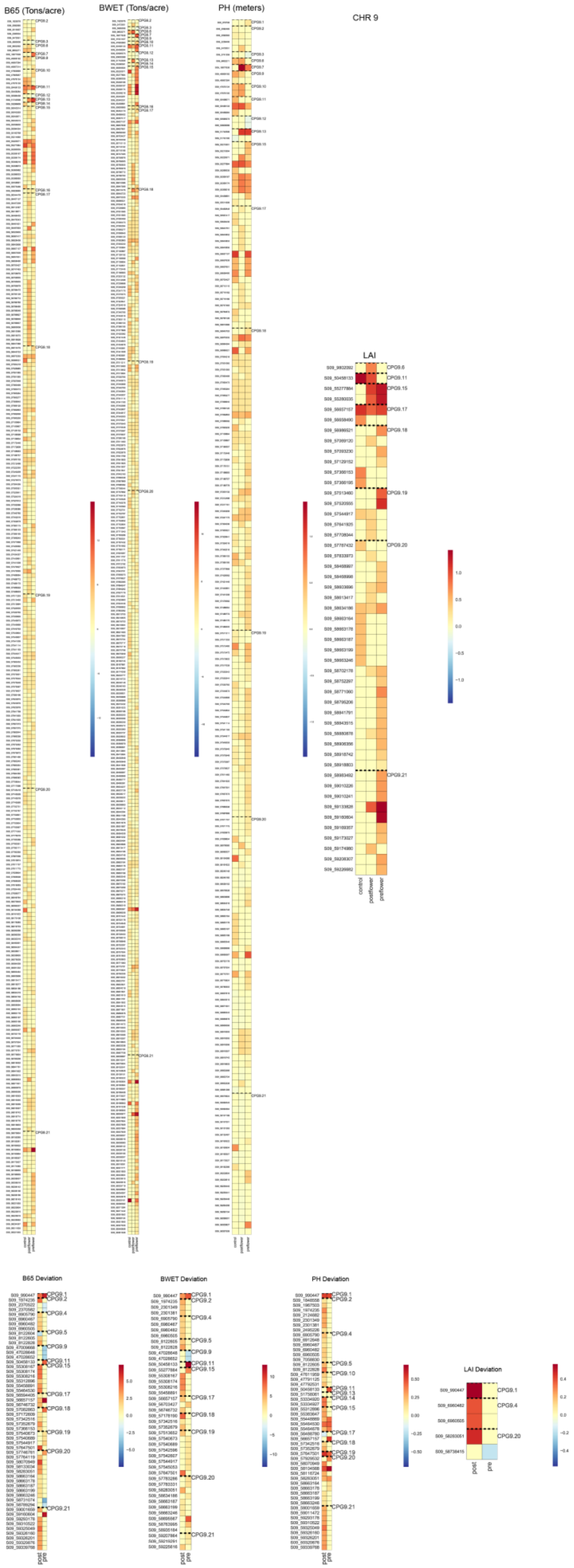

CHR 9
B65 (Tons/acre)
BWET (Tons/acre)
PH (meters)
LAI
control
postflower
preflower
B65 Deviation
BWET Deviation
PH Deviation
LAI Deviation
post
pre

Figure S9. Heat maps showing the average allele effects across locations and times of significant GWAS SNPs on each chromosome for all trait x treatment combinations. B65, BWET, PH, LAI (top rows) show results using raw data, B65 Deviation, BWET deviation, PH deviation, and LAI deviation (bottom rows) show results using the calculated differences between control and treatments where “preflower” = control – pre-flowering drought data and “postflower” = control – postflowering drought data. Note that each heat map has its own scale, but in all cases, darker red indicates that the minor allele confers an increase in the trait measurement (i.e., increased drought tolerance), darker blue indicates that the minor allele confers a decrease in the trait measure (i.e., decreased drought tolerance), and yellow indicates and effect at or close to 0. SNPs between dashed lines are in the same GWAS peak.

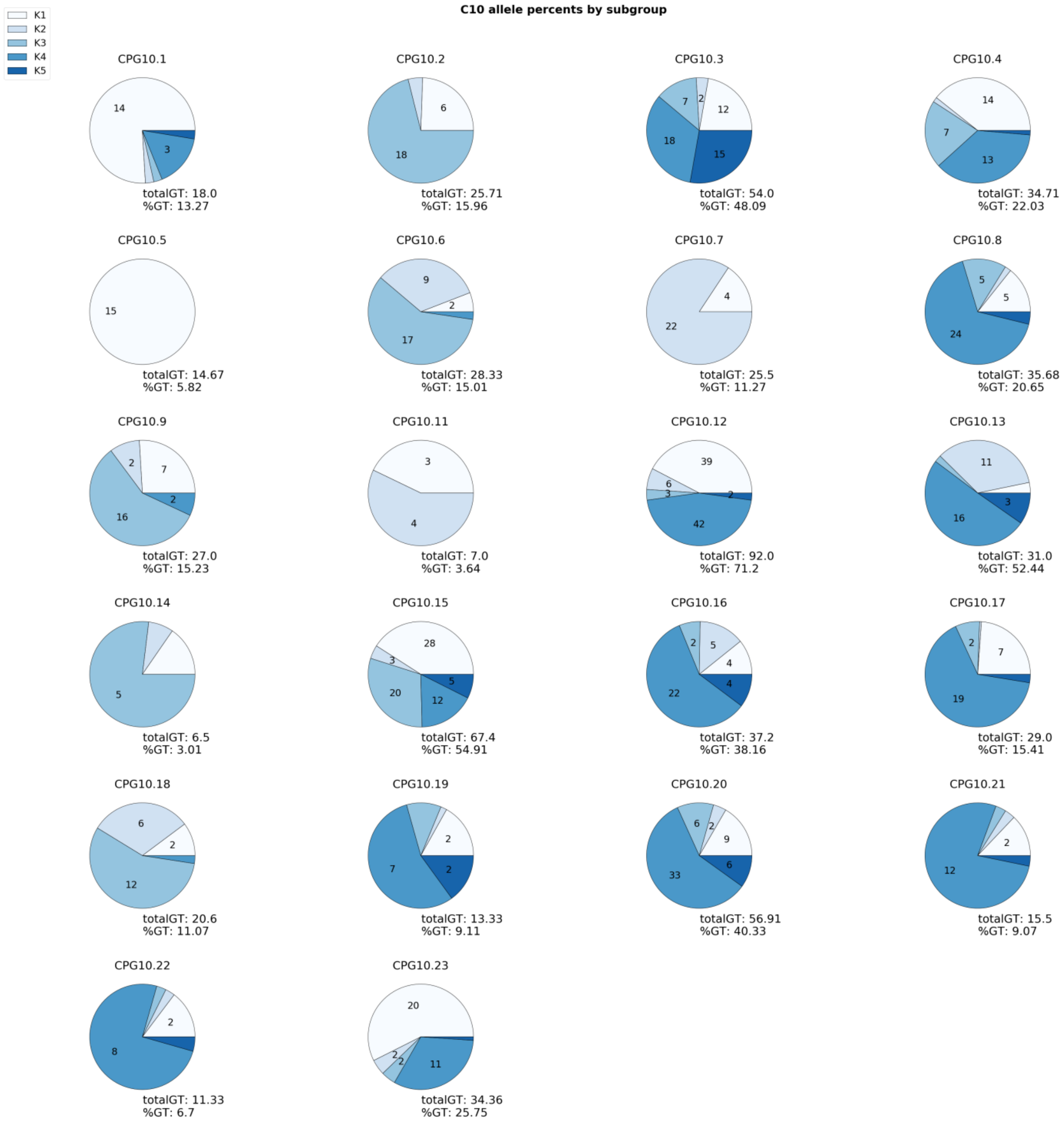
C10 allele percents by subgroup
K1
K2
K3
K4
K5
CPG10.1
totalGT: 18.0
%GT: 13.27
CPG10.2
totalGT: 25.71
%GT: 15.96
CPG10.3
totalGT: 54.0
%GT: 48.09
CPG10.4
totalGT: 34.71
%GT: 22.03
CPG10.5
totalGT: 14.67
%GT: 5.82
CPG10.6
totalGT: 28.33
%GT: 15.01
CPG10.7
totalGT: 25.5
%GT: 11.27
CPG10.8
totalGT: 35.68
%GT: 20.65
CPG10.9
totalGT: 27.0
%GT: 15.23
CPG10.11
totalGT: 7.0
%GT: 3.64
CPG10.12
totalGT: 92.0
%GT: 71.2
CPG10.13
totalGT: 31.0
%GT: 52.44
CPG10.14
totalGT: 6.5
%GT: 3.01
CPG10.15
totalGT: 67.4
%GT: 54.91
CPG10.16
totalGT: 37.2
%GT: 38.16
CPG10.17
totalGT: 29.0
%GT: 15.41
CPG10.18
totalGT: 20.6
%GT: 11.07
CPG10.19
totalGT: 13.33
%GT: 9.11
CPG10.20
totalGT: 56.91
%GT: 40.33
CPG10.21
totalGT: 15.5
%GT: 9.07
CPG10.22
totalGT: 11.33
%GT: 6.7
CPG10.23
totalGT: 34.36
%GT: 25.75

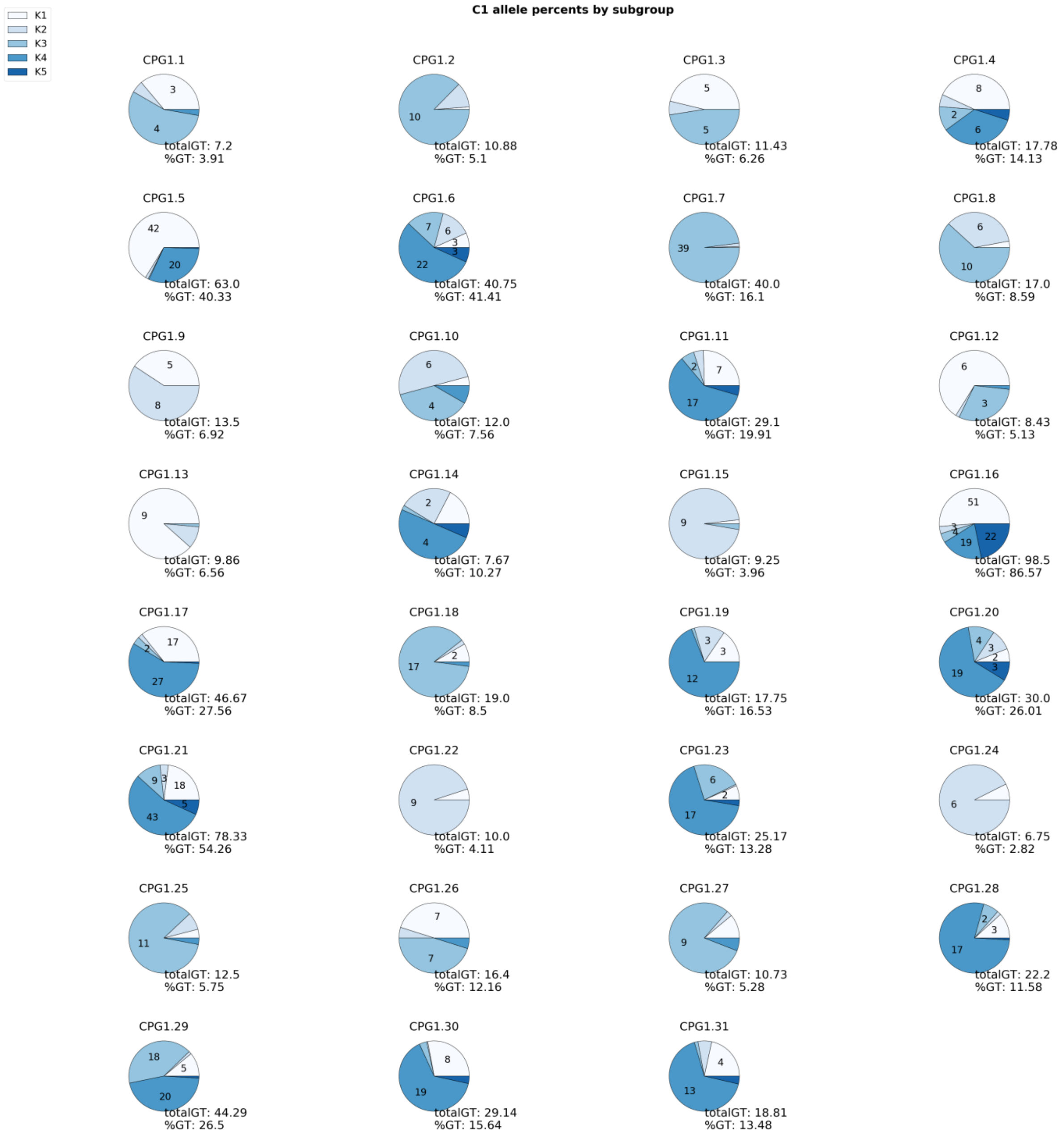
C1 allele percents by subgroup
K1
K2
K3
K4
K5
CPG1.1
3
4
totalGT: 7.2
%GT: 3.91
CPG1.2
10
totalGT: 10.88
%GT: 5.1
CPG1.3
5
5
totalGT: 11.43
%GT: 6.26
CPG1.4
8
2
6
totalGT: 17.78
%GT: 14.13
CPG1.5
42
20
totalGT: 63.0
%GT: 40.33
CPG1.6
7
6
3
3
22
totalGT: 40.75
%GT: 41.41
CPG1.7
39
totalGT: 40.0
%GT: 16.1
CPG1.8
6
10
totalGT: 17.0
%GT: 8.59
CPG1.9
5
8
totalGT: 13.5
%GT: 6.92
CPG1.10
6
4
totalGT: 12.0
%GT: 7.56
CPG1.11
2
7
17
totalGT: 29.1
%GT: 19.91
CPG1.12
6
3
totalGT: 8.43
%GT: 5.13
CPG1.13
9
totalGT: 9.86
%GT: 6.56
CPG1.14
2
4
totalGT: 7.67
%GT: 10.27
CPG1.15
9
totalGT: 9.25
%GT: 3.96
CPG1.16
51
22
19
totalGT: 98.5
%GT: 86.57
CPG1.17
17
2
27
totalGT: 46.67
%GT: 27.56
CPG1.18
2
17
totalGT: 19.0
%GT: 8.5
CPG1.19
3
3
12
totalGT: 17.75
%GT: 16.53
CPG1.20
4
3
2
3
19
totalGT: 30.0
%GT: 26.01
CPG1.21
9
3
18
5
43
totalGT: 78.33
%GT: 54.26
CPG1.22
9
totalGT: 10.0
%GT: 4.11
CPG1.23
6
2
17
totalGT: 25.17
%GT: 13.28
CPG1.24
6
totalGT: 6.75
%GT: 2.82
CPG1.25
11
totalGT: 12.5
%GT: 5.75
CPG1.26
7
7
totalGT: 16.4
%GT: 12.16
CPG1.27
9
totalGT: 10.73
%GT: 5.28
CPG1.28
2
3
17
totalGT: 22.2
%GT: 11.58
CPG1.29
18
5
20
totalGT: 44.29
%GT: 26.5
CPG1.30
8
19
totalGT: 29.14
%GT: 15.64
CPG1.31
4
13
totalGT: 18.81
%GT: 13.48

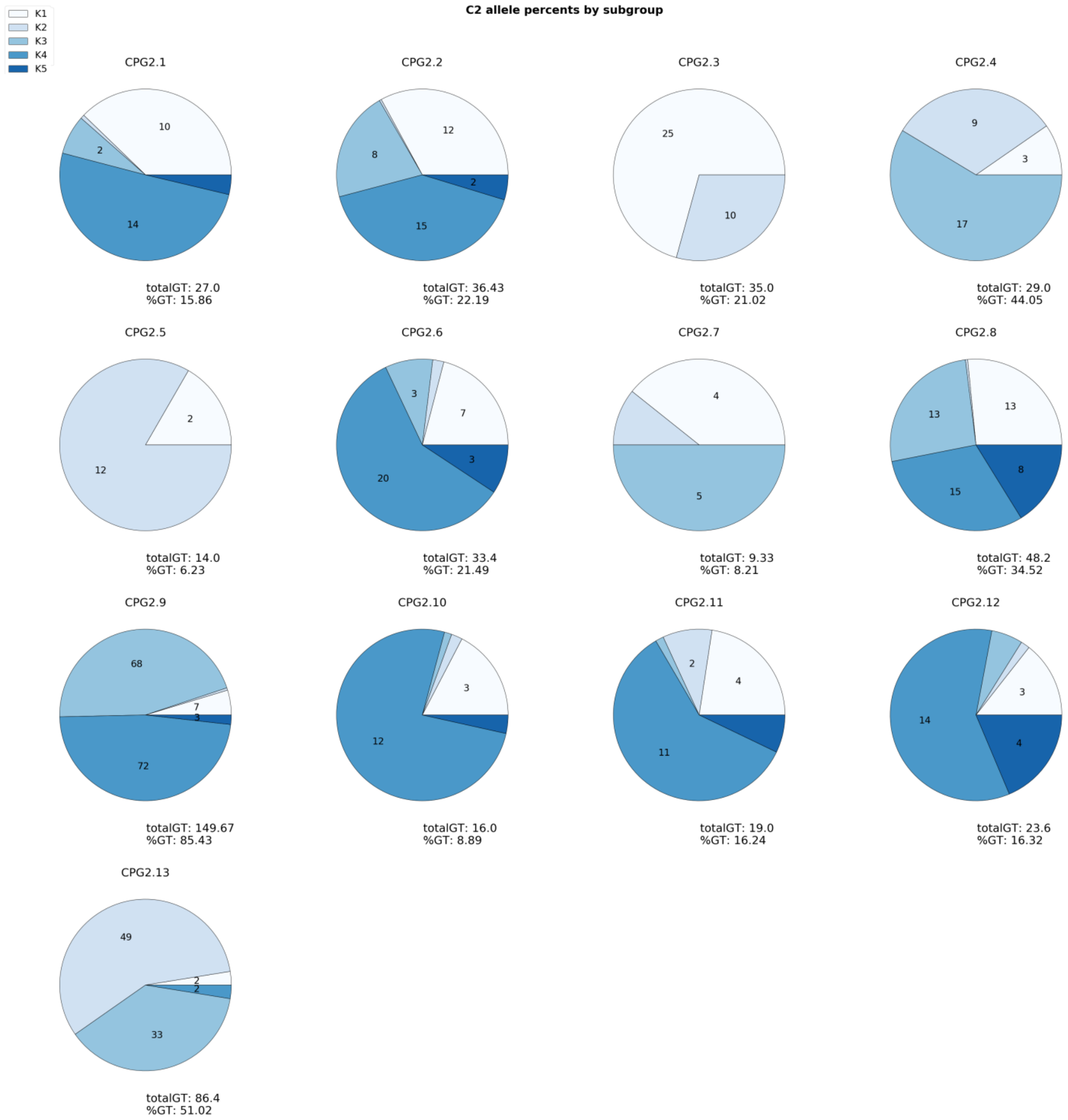
C2 allele percents by subgroup
K1
K2
K3
K4
K5
CPG2.1
10
2
14
totalGT: 27.0
%GT: 15.86
CPG2.2
12
8
2
15
totalGT: 36.43
%GT: 22.19
CPG2.3
25
10
totalGT: 35.0
%GT: 21.02
CPG2.4
9
3
17
totalGT: 29.0
%GT: 44.05
CPG2.5
2
12
totalGT: 14.0
%GT: 6.23
CPG2.6
3
7
3
20
totalGT: 33.4
%GT: 21.49
CPG2.7
4
5
totalGT: 9.33
%GT: 8.21
CPG2.8
13
13
8
15
totalGT: 48.2
%GT: 34.52
CPG2.9
68
7
3
72
totalGT: 149.67
%GT: 85.43
CPG2.10
3
12
totalGT: 16.0
%GT: 8.89
CPG2.11
2
4
11
totalGT: 19.0
%GT: 16.24
CPG2.12
3
14
4
totalGT: 23.6
%GT: 16.32
CPG2.13
49
2
2
33
totalGT: 86.4
%GT: 51.02

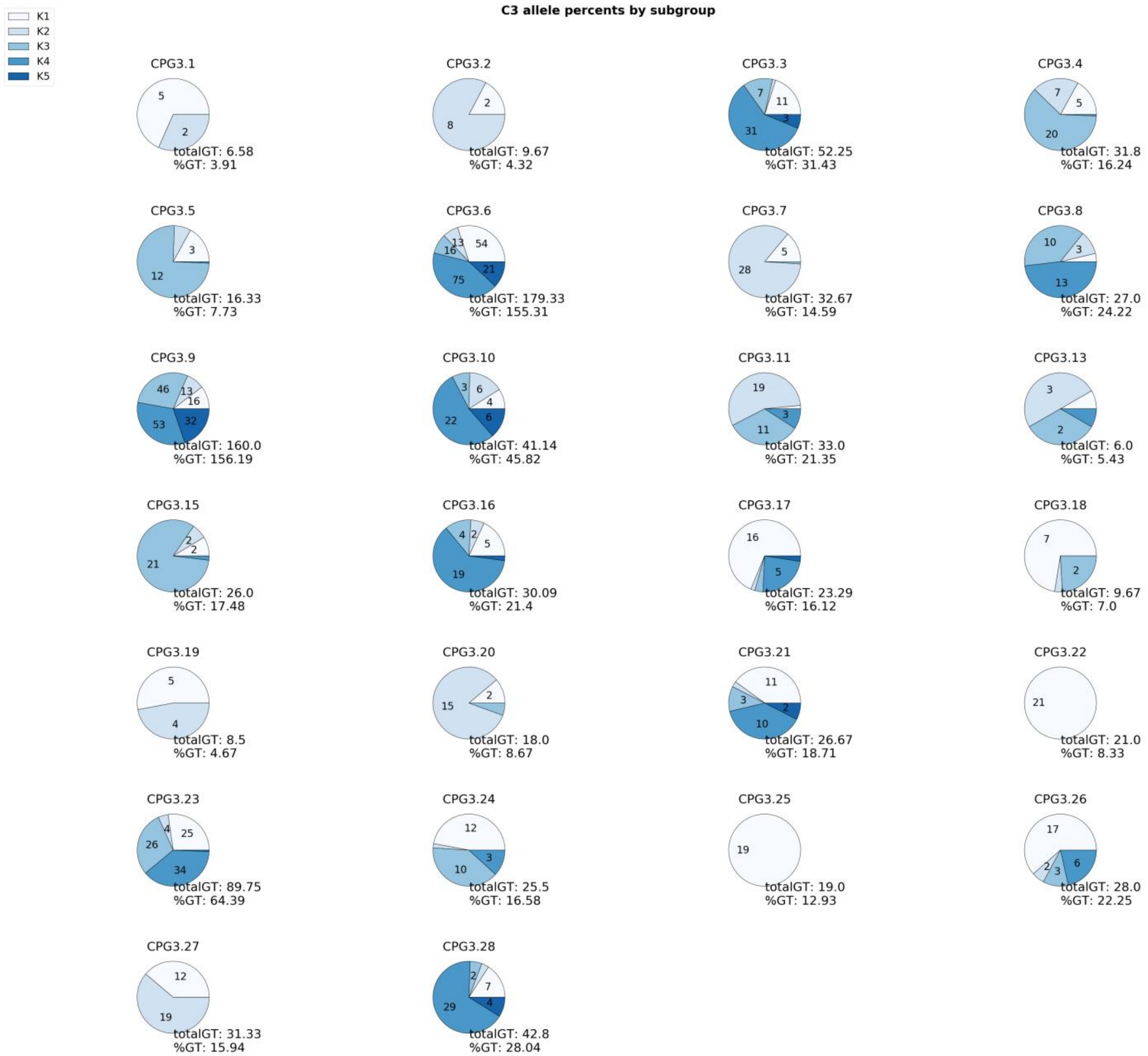
K1
K2
K3
K4
K5
C3 allele percents by subgroup
CPG3.1
totalGT: 6.58
%GT: 3.91
CPG3.2
totalGT: 9.67
%GT: 4.32
CPG3.3
totalGT: 52.25
%GT: 31.43
CPG3.4
totalGT: 31.8
%GT: 16.24
CPG3.5
totalGT: 16.33
%GT: 7.73
CPG3.6
totalGT: 179.33
%GT: 155.31
CPG3.7
totalGT: 32.67
%GT: 14.59
CPG3.8
totalGT: 27.0
%GT: 24.22
CPG3.9
totalGT: 160.0
%GT: 156.19
CPG3.10
totalGT: 41.14
%GT: 45.82
CPG3.11
totalGT: 33.0
%GT: 21.35
CPG3.13
totalGT: 6.0
%GT: 5.43
CPG3.15
totalGT: 26.0
%GT: 17.48
CPG3.16
totalGT: 30.09
%GT: 21.4
CPG3.17
totalGT: 23.29
%GT: 16.12
CPG3.18
totalGT: 9.67
%GT: 7.0
CPG3.19
totalGT: 8.5
%GT: 4.67
CPG3.20
totalGT: 18.0
%GT: 8.67
CPG3.21
totalGT: 26.67
%GT: 18.71
CPG3.22
totalGT: 21.0
%GT: 8.33
CPG3.23
totalGT: 89.75
%GT: 64.39
CPG3.24
totalGT: 25.5
%GT: 16.58
CPG3.25
totalGT: 19.0
%GT: 12.93
CPG3.26
totalGT: 28.0
%GT: 22.25
CPG3.27
totalGT: 31.33
%GT: 15.94
CPG3.28
totalGT: 42.8
%GT: 28.04

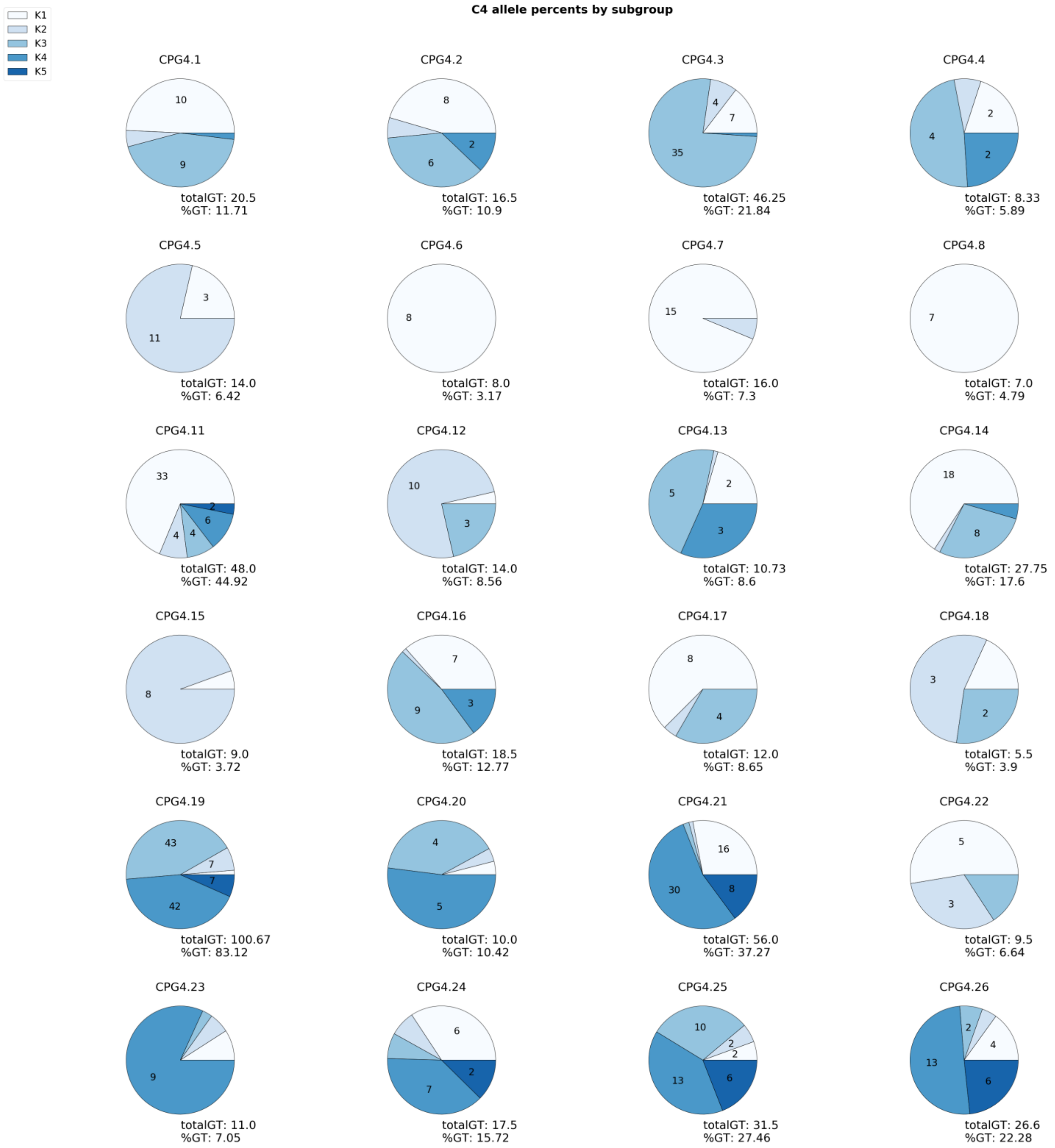
C4 allele percents by subgroup
K1
K2
K3
K4
K5
CPG4.1
totalGT: 20.5
%GT: 11.71
CPG4.2
totalGT: 16.5
%GT: 10.9
CPG4.3
totalGT: 46.25
%GT: 21.84
CPG4.4
totalGT: 8.33
%GT: 5.89
CPG4.5
totalGT: 14.0
%GT: 6.42
CPG4.6
totalGT: 8.0
%GT: 3.17
CPG4.7
totalGT: 16.0
%GT: 7.3
CPG4.8
totalGT: 7.0
%GT: 4.79
CPG4.11
totalGT: 48.0
%GT: 44.92
CPG4.12
totalGT: 14.0
%GT: 8.56
CPG4.13
totalGT: 10.73
%GT: 8.6
CPG4.14
totalGT: 27.75
%GT: 17.6
CPG4.15
totalGT: 9.0
%GT: 3.72
CPG4.16
totalGT: 18.5
%GT: 12.77
CPG4.17
totalGT: 12.0
%GT: 8.65
CPG4.18
totalGT: 5.5
%GT: 3.9
CPG4.19
totalGT: 100.67
%GT: 83.12
CPG4.20
totalGT: 10.0
%GT: 10.42
CPG4.21
totalGT: 56.0
%GT: 37.27
CPG4.22
totalGT: 9.5
%GT: 6.64
CPG4.23
totalGT: 11.0
%GT: 7.05
CPG4.24
totalGT: 17.5
%GT: 15.72
CPG4.25
totalGT: 31.5
%GT: 27.46
CPG4.26
totalGT: 26.6
%GT: 22.28

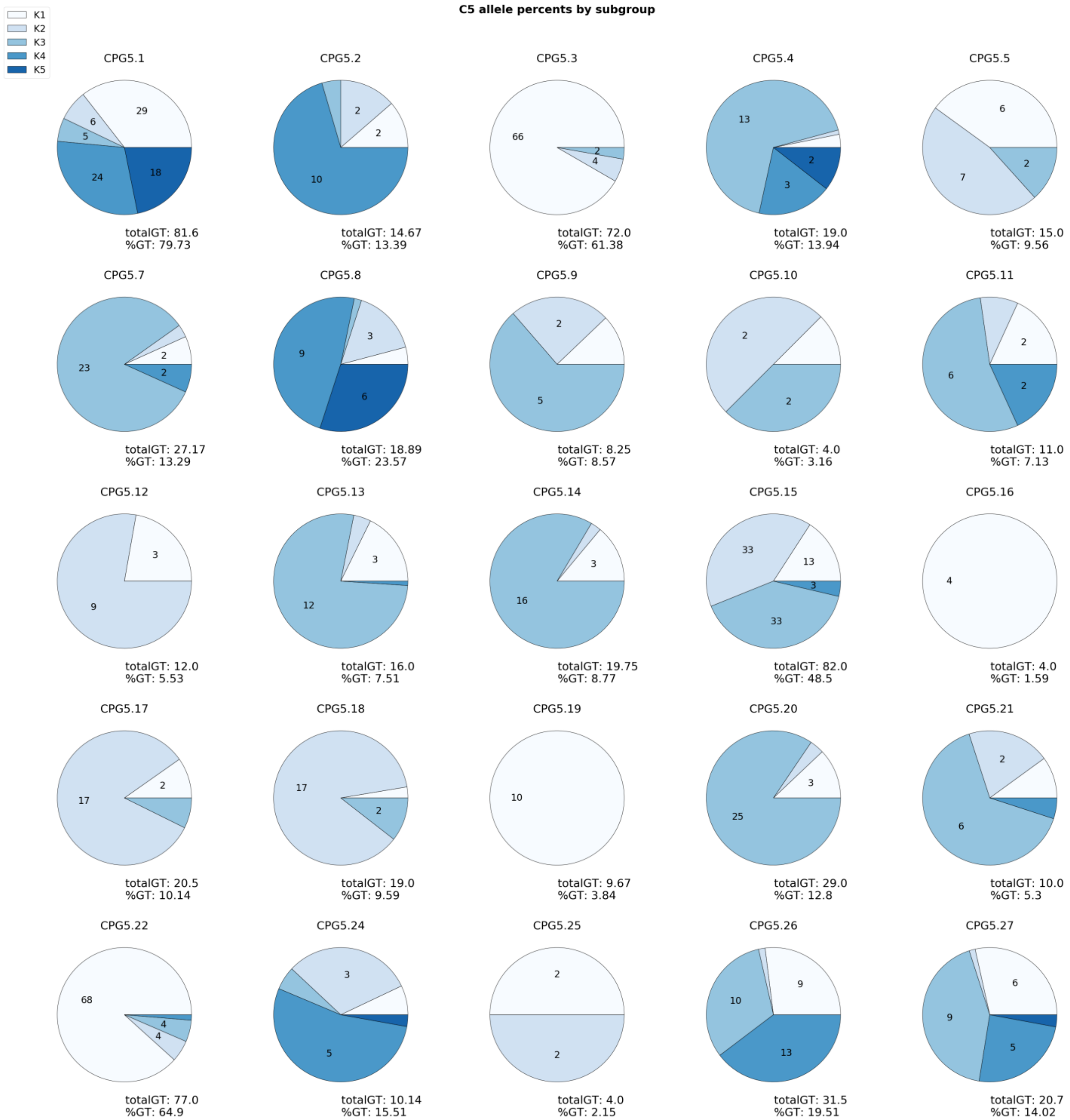
C5 allele percents by subgroup
K1
K2
K3
K4
K5
CPG5.1
29
6
5
24
18
totalGT: 81.6
%GT: 79.73
CPG5.2
2
2
10
totalGT: 14.67
%GT: 13.39
CPG5.3
66
2
4
totalGT: 72.0
%GT: 61.38
CPG5.4
13
2
3
totalGT: 19.0
%GT: 13.94
CPG5.5
6
7
2
totalGT: 15.0
%GT: 9.56
CPG5.7
23
2
2
totalGT: 27.17
%GT: 13.29
CPG5.8
3
9
6
totalGT: 18.89
%GT: 23.57
CPG5.9
2
5
totalGT: 8.25
%GT: 8.57
CPG5.10
2
2
totalGT: 4.0
%GT: 3.16
CPG5.11
2
6
2
totalGT: 11.0
%GT: 7.13
CPG5.12
3
9
totalGT: 12.0
%GT: 5.53
CPG5.13
3
12
totalGT: 16.0
%GT: 7.51
CPG5.14
3
16
totalGT: 19.75
%GT: 8.77
CPG5.15
33
13
3
33
totalGT: 82.0
%GT: 48.5
CPG5.16
4
totalGT: 4.0
%GT: 1.59
CPG5.17
2
17
totalGT: 20.5
%GT: 10.14
CPG5.18
17
2
totalGT: 19.0
%GT: 9.59
CPG5.19
10
totalGT: 9.67
%GT: 3.84
CPG5.20
3
25
totalGT: 29.0
%GT: 12.8
CPG5.21
2
6
totalGT: 10.0
%GT: 5.3
CPG5.22
68
4
4
totalGT: 77.0
%GT: 64.9
CPG5.24
3
5
totalGT: 10.14
%GT: 15.51
CPG5.25
2
2
totalGT: 4.0
%GT: 2.15
CPG5.26
9
10
13
totalGT: 31.5
%GT: 19.51
CPG5.27
6
9
5
totalGT: 20.7
%GT: 14.02

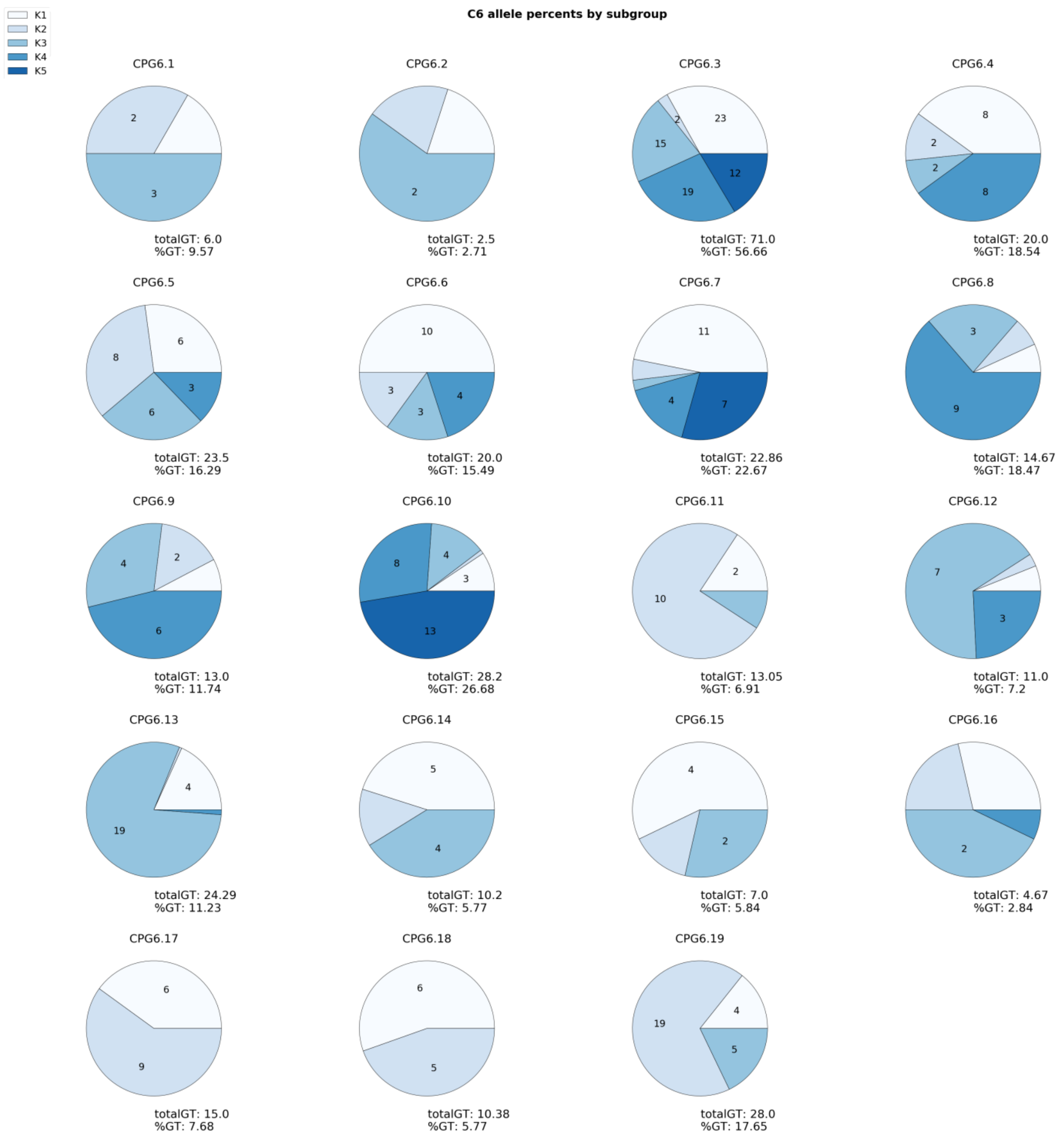

C6 allele percents by subgroup
K1
K2
K3
K4
K5
CPG6.1
2
3
totalGT: 6.0
%GT: 9.57
CPG6.2
2
totalGT: 2.5
%GT: 2.71
CPG6.3
2
23
15
12
19
totalGT: 71.0
%GT: 56.66
CPG6.4
8
2
2
8
totalGT: 20.0
%GT: 18.54
CPG6.5
6
8
3
6
totalGT: 23.5
%GT: 16.29
CPG6.6
10
3
4
3
totalGT: 20.0
%GT: 15.49
CPG6.7
11
4
7
totalGT: 22.86
%GT: 22.67
CPG6.8
3
9
totalGT: 14.67
%GT: 18.47
CPG6.9
2
4
6
totalGT: 13.0
%GT: 11.74
CPG6.10
8
4
3
13
totalGT: 28.2
%GT: 26.68
CPG6.11
2
10
totalGT: 13.05
%GT: 6.91
CPG6.12
7
3
totalGT: 11.0
%GT: 7.2
CPG6.13
4
19
totalGT: 24.29
%GT: 11.23
CPG6.14
5
4
totalGT: 10.2
%GT: 5.77
CPG6.15
4
2
totalGT: 7.0
%GT: 5.84
CPG6.16
2
totalGT: 4.67
%GT: 2.84
CPG6.17
6
9
totalGT: 15.0
%GT: 7.68
CPG6.18
6
5
totalGT: 10.38
%GT: 5.77
CPG6.19
4
19
5
totalGT: 28.0
%GT: 17.65

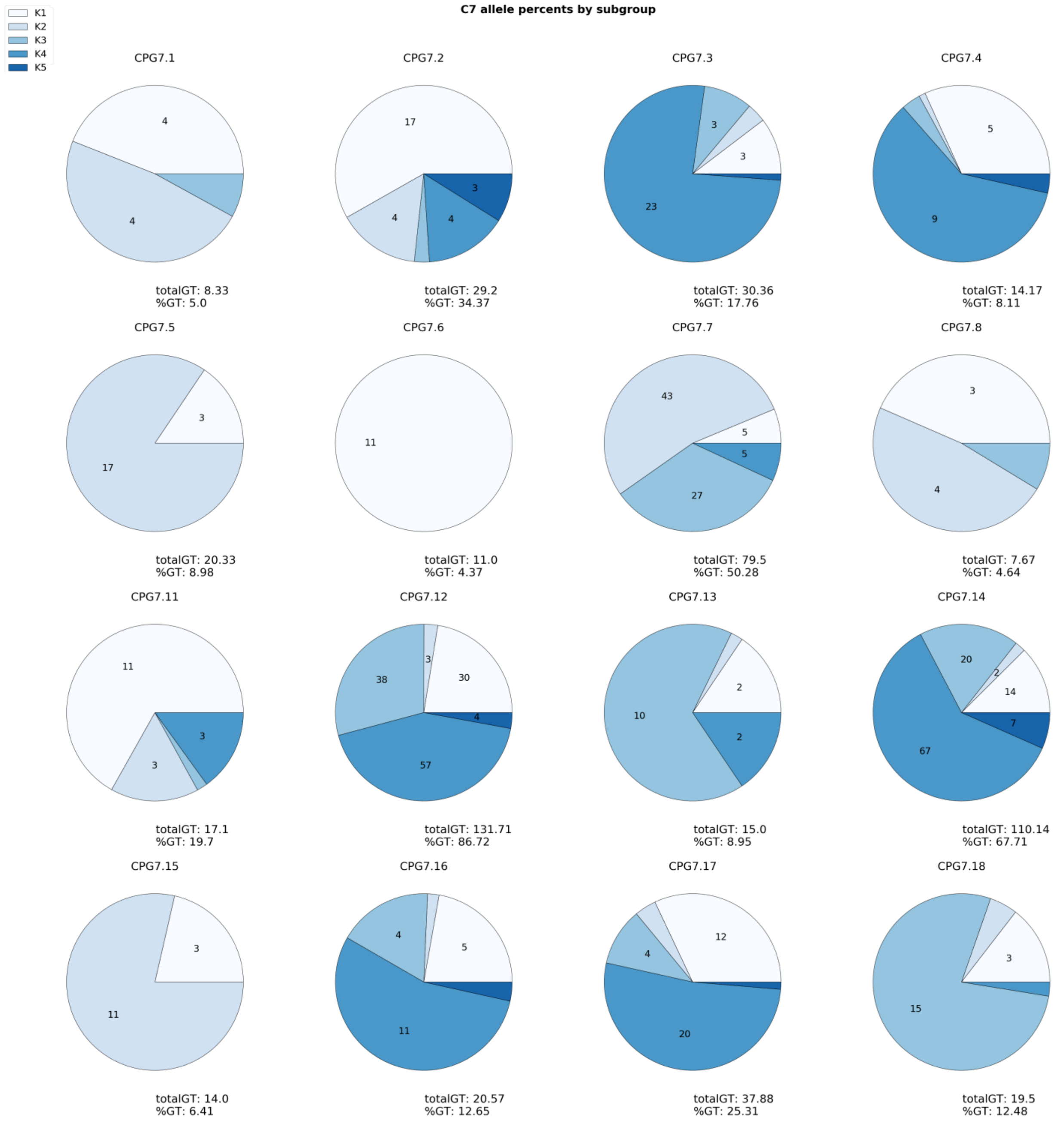
C7 allele percents by subgroup
K1
K2
K3
K4
K5
CPG7.1
4
4
totalGT: 8.33
%GT: 5.0
CPG7.2
17
4
4
3
totalGT: 29.2
%GT: 34.37
CPG7.3
3
3
23
totalGT: 30.36
%GT: 17.76
CPG7.4
5
9
totalGT: 14.17
%GT: 8.11
CPG7.5
3
17
totalGT: 20.33
%GT: 8.98
CPG7.6
11
totalGT: 11.0
%GT: 4.37
CPG7.7
43
5
5
27
totalGT: 79.5
%GT: 50.28
CPG7.8
3
4
totalGT: 7.67
%GT: 4.64
CPG7.11
11
3
3
totalGT: 17.1
%GT: 19.7
CPG7.12
3
38
30
4
57
totalGT: 131.71
%GT: 86.72
CPG7.13
2
10
2
totalGT: 15.0
%GT: 8.95
CPG7.14
20
2
14
7
67
totalGT: 110.14
%GT: 67.71
CPG7.15
3
11
totalGT: 14.0
%GT: 6.41
CPG7.16
4
5
11
totalGT: 20.57
%GT: 12.65
CPG7.17
12
4
20
totalGT: 37.88
%GT: 25.31
CPG7.18
3
15
totalGT: 19.5
%GT: 12.48

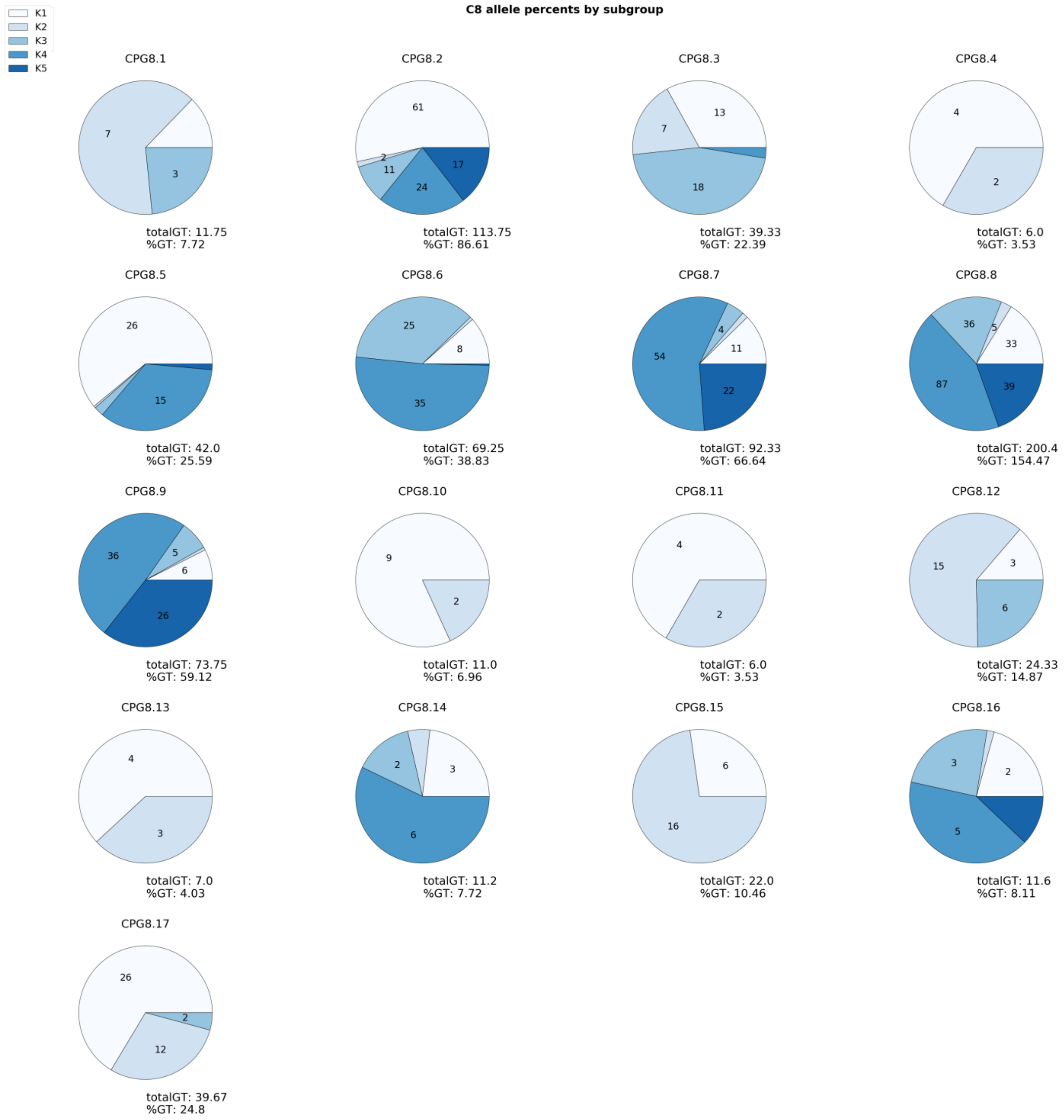
C8 allele percents by subgroup
K1
K2
K3
K4
K5
CPG8.1
7
3
totalGT: 11.75
%GT: 7.72
CPG8.2
61
2
11
24
17
totalGT: 113.75
%GT: 86.61
CPG8.3
13
7
18
totalGT: 39.33
%GT: 22.39
CPG8.4
4
2
totalGT: 6.0
%GT: 3.53
CPG8.5
26
15
totalGT: 42.0
%GT: 25.59
CPG8.6
25
8
35
totalGT: 69.25
%GT: 38.83
CPG8.7
4
11
54
22
totalGT: 92.33
%GT: 66.64
CPG8.8
36
5
33
87
39
totalGT: 200.4
%GT: 154.47
CPG8.9
36
5
6
26
totalGT: 73.75
%GT: 59.12
CPG8.10
9
2
totalGT: 11.0
%GT: 6.96
CPG8.11
4
2
totalGT: 6.0
%GT: 3.53
CPG8.12
15
3
6
totalGT: 24.33
%GT: 14.87
CPG8.13
4
3
totalGT: 7.0
%GT: 4.03
CPG8.14
2
3
6
totalGT: 11.2
%GT: 7.72
CPG8.15
6
16
totalGT: 22.0
%GT: 10.46
CPG8.16
3
2
5
totalGT: 11.6
%GT: 8.11
CPG8.17
26
2
12
totalGT: 39.67
%GT: 24.8

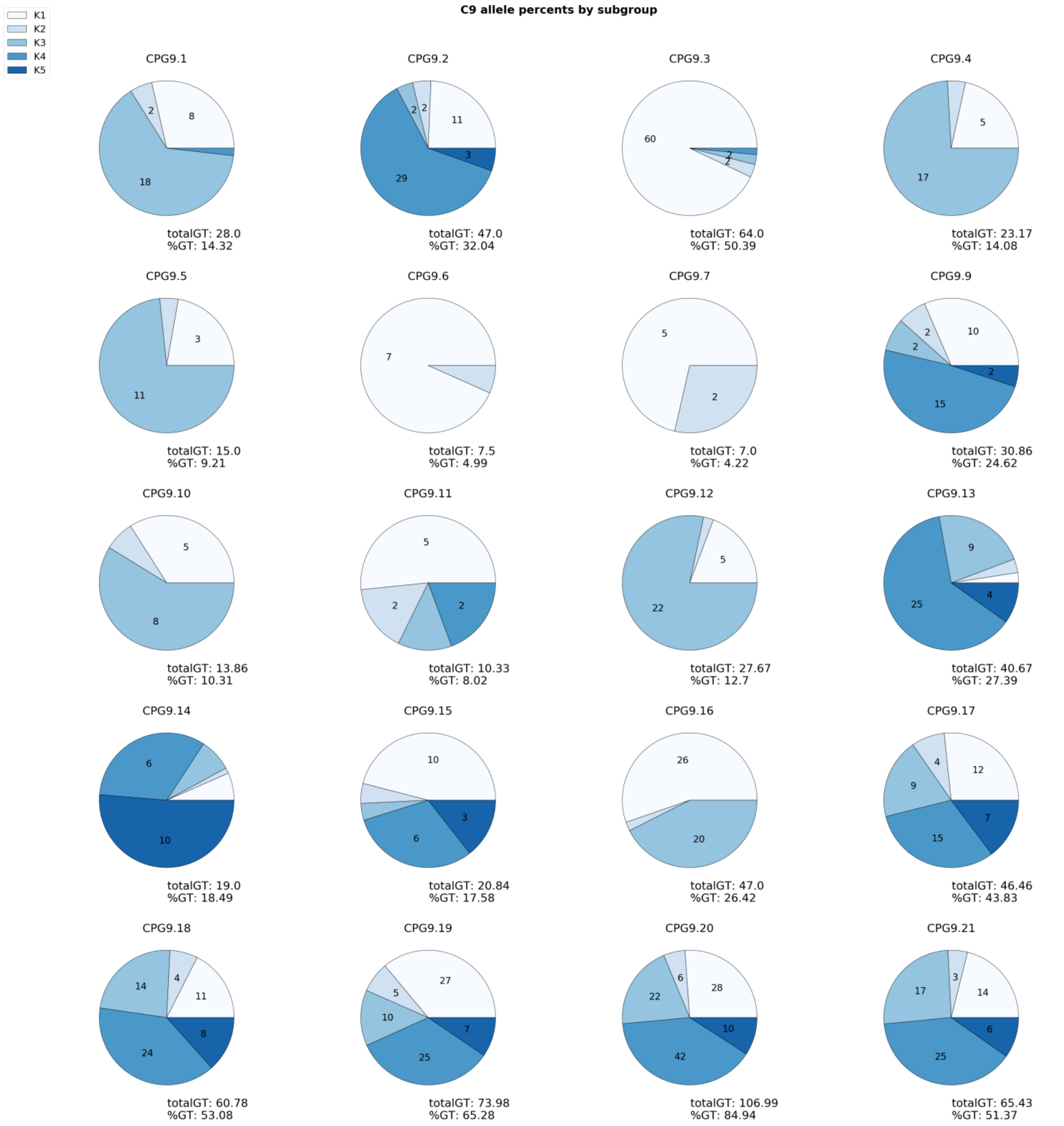

Figure S10. Pie plots showing the number and proportion of individuals in each subgroup (K 1-5) that carry the effect (minor) allele for a given GWAS peak, by chromosome.
